# Sphingolipid biosynthesis in man and microbes

**DOI:** 10.1039/c8np00019k

**Published:** 2018-06-04

**Authors:** Peter J. Harrison, Teresa M. Dunn, Dominic J. Campopiano

**Affiliations:** a School of Chemistry , University of Edinburgh , David Brewster Road , Edinburgh , EH9 3FJ , UK . Email: Dominic.Campopiano@ed.ac.uk; b Department of Biochemistry and Molecular Biology , Uniformed Services University , Bethesda , Maryland 20814 , USA

## Abstract

A new review covering up to 2018

Sphingolipids are essential molecules that, despite their long history, are still stimulating interest today. The reasons for this are that, as well as playing structural roles within cell membranes, they have also been shown to perform a myriad of cell signalling functions vital to the correct function of eukaryotic and prokaryotic organisms. Indeed, sphingolipid disregulation that alters the tightly-controlled balance of these key lipids has been closely linked to a number of diseases such as diabetes, asthma and various neuropathologies. Sphingolipid biogenesis, metabolism and regulation is mediated by a large number of enzymes, proteins and second messengers. There appears to be a core pathway common to all sphingolipid-producing organisms but recent studies have begun to dissect out important, species-specific differences. Many of these have only recently been discovered and in most cases the molecular and biochemical details are only beginning to emerge. Where there is a direct link from classic biochemistry to clinical symptoms, a number a drug companies have undertaken a medicinal chemistry campaign to try to deliver a therapeutic intervention to alleviate a number of diseases. Where appropriate, we highlight targets where natural products have been exploited as useful tools. Taking all these aspects into account this review covers the structural, mechanistic and regulatory features of sphingolipid biosynthetic and metabolic enzymes.

## Abstract 2

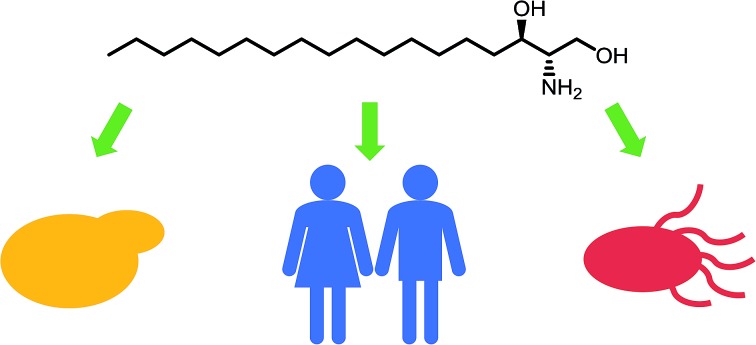
Sphingolipids are found in nearly all domains of life where they play a myriad of essential roles in structure and signalling. This review covers recent highlights from studies of the structures, mechanisms and inhibitors of key enzymes from the sphingolipid biosynthetic enzymes of prokaryotes and eukaryotes.

## 1 Introduction

The chemistry and biology of sphingolipids (SLs) is a complicated field of research that requires an understanding of protein chemistry, enzyme kinetics, cellular and structural biology, proteomics, lipidomics and pathogenesis to fully appreciate the fundamental roles that these molecules play across eukaryotic and some prokaryotic species. Despite first being identified over 130 years ago, the myriad roles of SLs in biology are only now beginning to be understood.^1^ SLs are part of a larger lipid family^2^ and are essential structural components of cell membranes and important signalling molecules that have been implicated in a wide variety of different cellular processes such as cell differentiation, pathogenesis and apoptosis.^3^–^5^ It is not surprising, therefore, that we are still discovering new roles of SLs today.

**Fig. 1:**
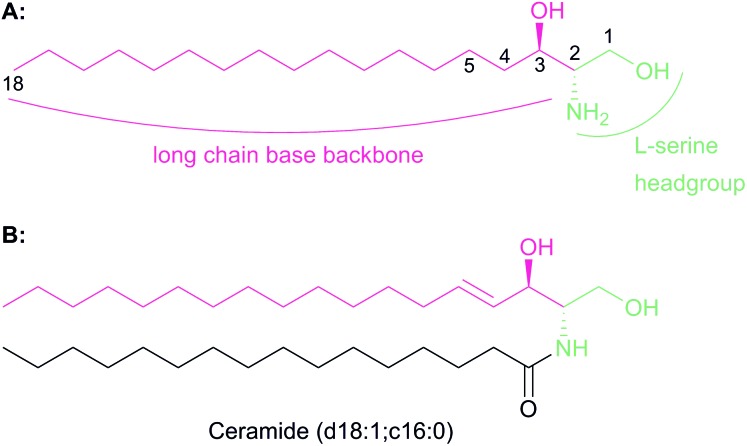
(A) Chemical structures of the sphingoid base backbone, which forms the core of all SLs. The l-serine derived moiety is highlighted in green and the fatty acyl-CoA moiety is highlighted in pink. The C1 and C2 hydroxyl and amines, the sites of further modification which generates complex SLs, are also highlighted. (B) *N*-Acylation of the sphingoid base generates ceramide. Shown is atypical ceramide derived from palmitoyl-CoA and *N*-acylation with C_16_-CoA (d18:1; c16:0). Further complexity is added by addition of head groups to the C1 hydroxyl.

All SLs are derived from l-serine and a fatty acid that together define a “sphingoid” base, also referred to as a long chain base (LCB) (Fig. 1a). Diversity in SLs is generated through variations in the fatty acid and the C1 head group and can be further expanded by acylation of the C2 amine by fatty acids of varying chain length. *N*-Acylation generates the ceramides (Fig. 1b, showing C_16_ ceramide, which can be considered as the base unit for SLs) and, for example, in plants, a combination of nine different LCBs with thirty two different fatty acids gives rise to a pool of 288 possible different ceramides.^6^ A vast, extensive reservoir of SLs and ceramides is possible through attachment of different head groups such as sugars, phosphate, fatty acid, phosphoinositol or phosphocholine to the C1 hydroxyl.^7^ This variation generates potentially well over 1000 structurally distinct SLs. As a result of these variations, the SL profile of organisms varies between taxa and indeed between species. For example, plants and yeast produce a sphingoid base, phytosphingosine, which contains a C4 hydroxyl, which is not abundant in mammals. Similarly the yeast *Saccharomyces cerevisiae* produces phosphoinositol containing complex SLs whilst both phosphoinositol and glucosylceramide-containing SLs are found in *Pichia pastoris*.^8^,^9^ This SL pool, known as the “sphingolipidome”, is in constant dynamic flux and is subject to the metabolic demands on the cell; a balance is struck between *de novo* biosynthesis and breakdown/recycling. SL homeostasis is maintained through complex regulatory pathways, the details of which are only now being understood.^10^

**Fig. 2:**
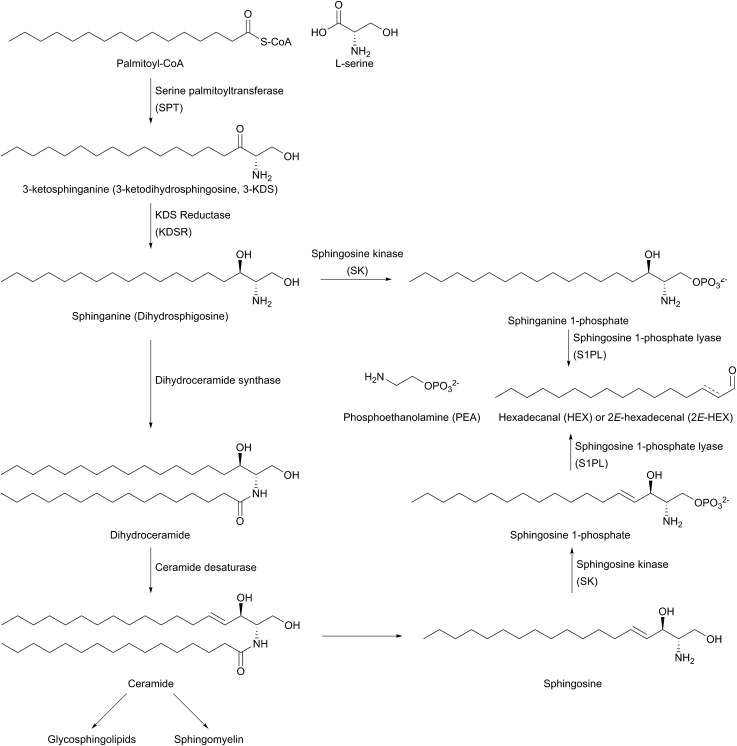
General overview of the SL biosynthesis pathway. There are, however, variations in the different types of sphingoid bases and ceramides produced across different organisms. As such, this general overview omits details such as C4 hydroxylation to produce phytosphingosine in plants, yeast and fungi.

The biosynthesis of SLs can be thought of as being split up into three parts (Fig. 2, which gives a general overview of SL biosynthesis). These three parts consist of the biosynthesis of sphingoid bases, which is then followed by the biosynthesis of ceramides (by the attachment of an amide linked fatty acid) and finally, formationation of complex SLs (through the attachment of head groups to C1 of the sphingoid base). SL biosynthesis and metabolism is intriguing in that, in all organisms studied to date, the pathway is bookended by two pyridoxal 5′-phosphate (PLP) dependent enzymes (Fig. 2); serine palmitoyltransferase (SPT) and sphingosine 1-phosphate lyase (S1PL). The *de novo* pathway begins with SPT, which catalyses the decarboxylative, Claisen-like condensation of l-serine with palmitoyl coenzyme-A (palmitoyl-CoA) to form 3-ketosphinganine (3-keto-dihydrosphingosine, 3-KDS). This SPT-catalysed reaction defines the 2*S*,3*R* (d-*erythro* diastereomer) stereochemistry of all downstream SLs and ceramides. The breakdown of SLs requires an LCB phosphate lyase (LCB1PL more commonly known as called S1PL), which performs a retro-aldol like cleavage of LCB 1-phosphate to phosphoethanolamine (PEA) and a long chain aldehyde (*e.g.* 2*E*-hexadecanal (2*E*-HEX)).^11^,^12^ In eukaryotes the majority of SL biosynthesis begins in the endoplasmic reticulum (ER).^7^,^13^ Thereafter, complex SL biosynthesis primarily occurs in the Golgi apparatus (although some SLs, for example sphingomyelin, can also be made in the plasma membrane).^14^,^15^ In addition to the *de novo* synthesis of SLs detailed above, SLs can also be formed from the turnover and recycling of LCBs and ceramides, which can then be fed back into the SL pool.^11^,^12^

SL levels are tightly controlled in cells. However, the molecular details by which SL levels are regulated are not currently understood in great detail. Disruption of SL homeostasis can give rise to disease states.^16^,^17^ Mutations to enzymes in the SL biosynthesis pathway also give rise to disease. For example, hereditary sensory and autonomic neuropathy type 1 (HSAN1) results from the formation of deoxysphingolipids (deoxy-SLs) which cannot be removed from the SL pool by the LCB1P lyase dependent pathway.^17^,^18^ The formation of these deoxy-SLs and their potential role as biomarkers for disease makes them of pharmaceutical interest.

The biological role of SLs and the complex interplay of signalling pathways that occurs in SL signalling is beyond the scope of this review. However, understanding the roles of SLs and the SL rheostat is a rapidly developing area of research. Several excellent recent review articles provide more information on the structure and function of SLs and the regulation of SLs.^6^,^8^,^9^ Readers are particularly directed to Alfred Merrill's 2011 seminal review of SLs which provides an excellent overview of SL structure and function.^7^,^19^ Here we will focus on the enzymes of SL biosynthesis and breakdown which have been structurally characterised and have been the target of medicinal chemistry campaigns by various pharmaceutical companies. A complete list of the enzymes discussed in this review, along with their associated UniProt and PDB codes is provided in Table 1. We also want to emphasise that SLs are also found in some prokaryotes and other higher organisms and we will discuss examples from across the phylogenetic tree of life.

**Table 1:** UniProt and PDB codes for characterised enzymes discussed in this review (references refer to the PDB entries)

Enzyme	Organism	UniProt code(s)	PDB code(s)	Ref.
SPT	*Sphingomonas paucimobilis*	Q93UV0	2JG2, ; 2JGT, ; 2W8J, ; 2W8T, ; 2W8U, ; 2W8V, ; 2W8W, ; 2XBN	39, 59 and 153
SPT	*Sphingomonas multivorum*	A7BFV6	3A2B	58
SPT	*Sphingomonas spiritivorum*	A7BFV7	—	
SPT	*Sphingomonas wittichii*	A5VD79	2X8U	54
SPT	*Bdellovibrio stolpii*	A7BFV8	—	
SPT	*Bacteroides fragilis*	Q5LCK4	—	
SPT	*Porphyromonas gingivalis*	Q7MTZ6	—	
SPT	*Saccharomyces cerevisiae*	P25045 (LCB1), P40970 (LCB2)	—	
SPT	*Homo sapiens*	O15269 (HsSPT1), O15270 (HsSPT2), Q9NUV7 (HsSPT3)	—	
SPT	*Toxoplasma gondii*	B9PTE5 (TgSPT1), B9QEB0 (TgSPT2)	—	
SPT	*Mus musculus*	O35704 (SPT1), P97363 (SPT2)	—	
ssSPT	*Saccharomyces cerevisiae*	Q3E790 (Tsc3p)	—	
ssSPT	*Homo sapiens*	Q969W0 (ssSPTa), Q8NFR3 (ssSPTb)	—	
ORMDL	*Homo sapiens*	Q9P0S3 (ORMDL1), Q53FV1 (ORMDL2), Q8N138 (ORMDL3)	—	
ORM	*Saccharomyces cerevisiae*	P53224 (ORM1), Q06144 (ORM2)	—	
KDSR	*Saccharomyces cerevisiae*	P38342	—	
KDSR	*Candida albicans*	Q59RQ2	—	
KDSR	*Aspergillus fumigatus*	Q4WSZ0	—	
KDSR	*Homo sapiens*	Q06136	—	
SK	*Homo sapiens*	Q9NYA1 (SK1), Q9NRA0 (SK2)	3VZC, ; 3VZD, ; 3VZB, ; 4L02, ; 4V24	237, 249 and 250
SK	*Saccharomyces cerevisiae*	Q12246 (LCB4), Q06147 (LCB5)	—	
S1PL	*Saccharomyces cerevisiae*	Q05567 (Dp11p)	3MC6,	271
S1PL	*Homo sapiens*	O95470	4Q6R	284
S1PL	*Burkholderia pseudomallei*	Q63IP8 (S1PL2021), Q63IP4 (S1PL2025)	5K1R	278
S1PL	*Symbiobacterium thermophilum*	Q67PY4	3MBB, ; 3MAU, ; 3MAF, ; 3MAD, ; 5EUE, ; 5EUD	271 and 285
S1PL	*Legionella pneumophila*	Q5ZTI6	4W8I	276

## 2 Serine palmitoyltransferase (SPT)

The first step in *de novo* SL biosynthesis is the condensation of l-serine with palmitoyl-CoA, catalysed by serine palmitoyltransferase (SPT), to form 3-KDS, the sphingoid base which is the starting point for all SLs (Fig. 2).^20^–^22^ The role of SPT as the first enzyme is conserved across all organisms studied to date. However, there are important differences in SPT biochemistry between taxa, one of the most significant being the subcellular localisation of the enzyme – in bacteria the enzyme is cytoplasmic, whereas in yeast, plants and mammals the enzyme is localised to the ER.^23^

### 2.1 Enzymology of SPT

SPT is a PLP-dependent enzyme, one of the broadest superfamilies of enzymes. PLP enzymes catalyse a wide variety of chemical reactions including C–C bond formation (8-amino-7-oxononanoate synthase (AONS)),^24^ C–C bond cleavage (serine hydroxymethyltransferase),^25^ racemisation (alanine racemase),^26^ transamination (aspartate aminotransferase),^27^ decarboxylation (3,4-dihydroxyphynlalanine decarboxylase)^28^ and dehydration (threonine dehydratase).^29^ Their substrates tend to be amino acids but they also play important roles in essential pathways such as cell wall^30^ and vitamin biosynthesis,^24^ antibiotic resistance^31^ and neurotransmitter metabolism.^28^ PLP enzymes display similarities in amino acid sequence, 3D structural fold and enzyme mechanism. SPT catalyses the formation of 3-KDS, an α-oxoamine and thus is part of the α-oxoamine synthase (AOS) family that catalyses Claisen like condensation reactions between amino acid and acyl-CoA substrates. The PLP superfamily has been sub-classified according to 3D structure, and sequence analysis places all SPTs characterised to date in the type-1 subfamily of PLP-dependent enzymes^32^–^34^ The catalytic mechanism of type-1 PLP enzymes is heavily influenced by the way the enzyme binds the PLP cofactor and its substrates within the catalytic fold. The best characterised AOS members are 5-aminolevulinate synthase (ALAS, heme biosynthesis),^35^ AONS (biotin biosynthesis),^24^ 2-amino-3-ketobutyrate coenzyme A ligase (KBL, l-threonine degradation)^36^ and CqsA (quorum sensing molecule biosynthesis).^37^ Although the extent to which the mechanism is conserved across AOS superfamily members is unknown, by using mechanistic data from across the family a catalytic mechanism for SPT was proposed.^38^ Evidence for these proposals has been obtained by the recent structural and mechanistic studies on the soluble, cytoplasmic bacterial SPTs from *Sphingomonas paucimobilis* and *Sphingomonas multivorum*.^39^–^43^

**Fig. 3:**
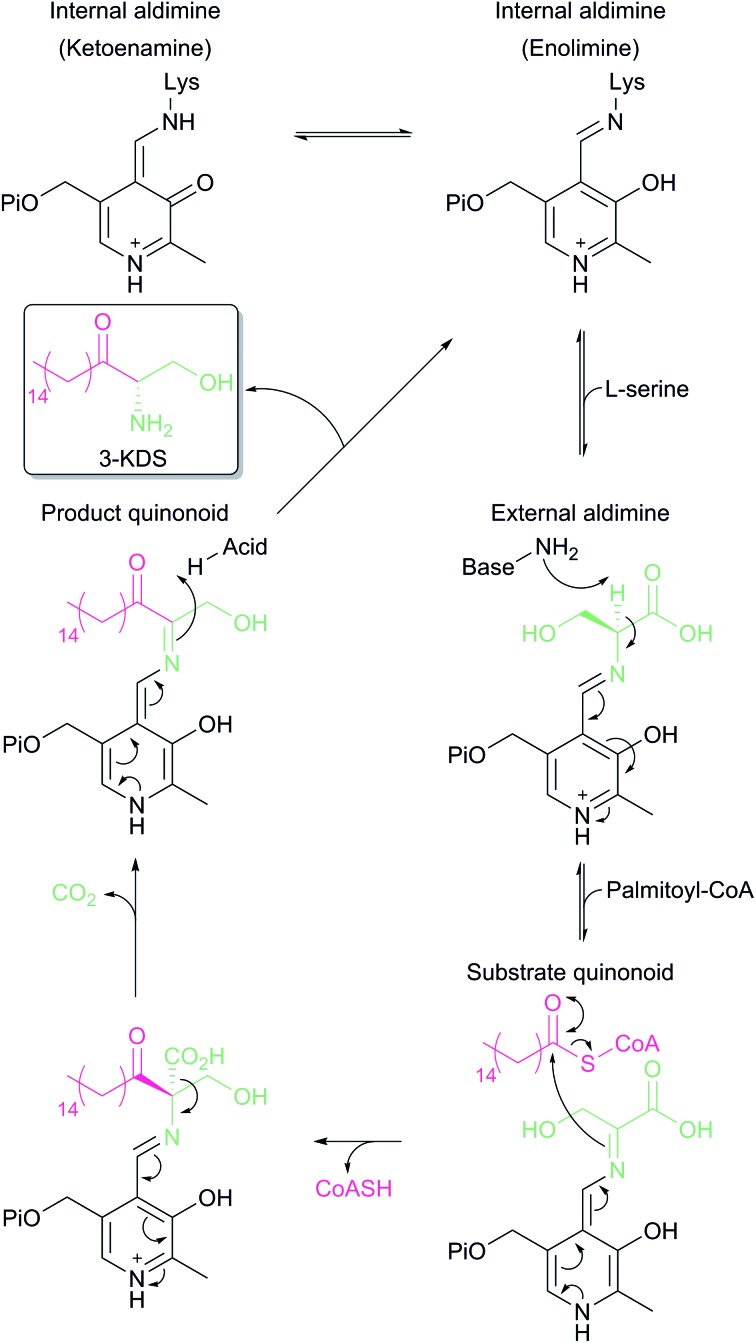
Proposed catalytic mechanism for SPT based on studies performed on *Sphingomonas* SPT and by analogy to other AOS family members. Briefly, binding of l-serine displaces PLP bound as an internal aldimine (Schiff's base) to the conserved active site lysine to form a PLP:l-serine external aldimine. Acyl-CoA binding causes abstraction of the α-proton from l-serine. Electron rebound onto the acyl-CoA thioester forms the C–C bond and releases free CoASH. Subsequent decarboxylation and displacement by the lysine side chain releases the 3-KDS product and re-forms the internal aldimine.

In the resting state of the prokaryotic SPT enzyme the PLP cofactor is bound to the enzyme at an active site lysine through a Schiff base linkage (Fig. 3). This can be observed spectrophotometrically through two characteristic absorbance maxima at 333 nm and 420 nm (corresponding to the enolimine and ketoenamine tautomers of PLP-Lys respectively) and is referred to as the internal aldimine.^44^,^45^ The SPT reaction begins with the binding of l-serine to the active site, which displaces lysine from PLP to form the PLP:l-Ser external aldimine complex. This is characterised by a decrease in absorbance at 333 nm with a concurrent increase at 425 nm (with the absorbance maxima shifting slightly from 420 nm in the internal aldimine). Following formation of the external aldimine, the second substrate (the palmitoyl-CoA thioester) binds, which is thought to cause a conformational change. This brings the proposed base – the PLP-binding lysine – in close enough proximity to allow proton abstraction from Cα and the formation of a quinonoid/carbanion intermediate. Electron rebound from the PLP cofactor then allows for nucleophilic attack on the palmitoyl-CoA thioester and stereoselective C–C bond formation. Following this, decarboxylation occurs readily from the β-keto acid intermediate, forming the PLP:3-KDS external aldimine product by abstracting an active site proton. Experiments with [2,3,3-^2^H_3_]-l-serine have shown that the α proton of serine is not retained in the 3-KDS product, implying that either the active site proton used to form the product 3-KDS is derived from bulk water, or that the initially abstracted proton is rapidly exchanged with bulk water.^46^ The 3-KDS is subsequently displaced from the PLP:3-KDS complex by the active site lysine, releasing the 3-KDS and allowing another round of catalysis to begin.

For some time, there was debate as to the true course of the SPT reaction. Did decarboxylation occur first, forming a quinonoid which then condensed onto the palmitoyl-CoA, or did α-proton deprotonation occur first? Experiments on α-oxamine family members (AONS, ALAS and SPT) whereby the α-proton is not retained in the final product indicate that the α-proton abstraction is the first step.^46^–^48^ Additional experiments on ALAS and AONS also lend support to the deprotonation-first mechanism.^49^,^50^ More direct proof for deprotonation as the first step was provided by an elegant set of experiments performed by Ikushiro and colleagues.^43^ A modified palmitoyl-CoA substrate analogue was synthesised, in which a methylene bridge was inserted between the sulphur atom and the carbonyl, to form an acyl-thioether (*S*-(2-oxoheptadecyl)-CoA). This subtle modification allows the analogue to bind to the SPT PLP:l-serine external aldmine complex and for the presumed conformational change to occur that would allow deprotonation from Cα. However, the thioether prevents cleavage of the acyl moiety from CoA, thus trapping the PLP enzyme complex in a substrate-activated state. Ikushiro then used ^1^H NMR to monitor proton signals of l-serine during the course of the reaction. The addition of l-serine to SPT (formation of the external aldimine) did not alter the chemical shift of the l-serine protons. However, the subsequent addition of the acyl-thioether analogue caused a change in the chemical shifts consistent with α-deprotonation (forming the quinonoid intermediate) at ∼100-fold increased rate. This quinonoid intermediate was observed spectrophotometrically by the formation of a peak at 490 nm, which is stable for several hours at room temperature. Consequently, palmitoyl-CoA binding is a prerequisite for α-deprotonation and subsequent attack onto the thio-ether of palmitoyl-CoA.^40^

### 2.2 SPT in bacteria

SLs are not commonly found as components of either Gram positive or negative bacterial membranes. However, SLs have been identified in a select group of microbes, including the *Bacteroides* and *Sphingomonads*.^51^,^52^ The purification of a bacterial SPT from crude cell lysate was first reported by Lev & Milford in 1981, who purified an enzyme with what they referred to as “3-ketodihydrosphingosine synthetase” activity from *Bacteroides melaninogenicus* >100 fold from cell free extracts.^53^ This *B. melaninogenicus* SPT demonstrated a preference for palmitoyl-CoA, but could utilise a variety of different acyl-CoA substrates, from as short as decanoyl-CoA (C_10_) to as long as oleoyl-CoA (C_18_).

The first purification of a bacterial SPT to homogeneity was reported by Ikushiro *et al.* in 2001 from *S. paucimobilis*, which also allowed the encoding gene to be cloned and sequenced (SpSPT, Q93UV0, Table 1).^44^ The SpSPT displays 30% homology with other AONS family members and contains the conserved PLP binding lysine residue (Lys265). Recombinant SpSPT isolated from *E. coli* is a soluble homodimer and binds one molecule of PLP per subunit, and also exhibits a tolerance to a range of acyl-CoA substrates, similar to *B. melaninogenicus* SPT*.* Interestingly, when expressed heterologously in *E. coli*, the host produces the 3-KDS product from endogenous l-serine and palmitoyl-CoA (Gable *et al.*, unpublished).

**Fig. 4:**
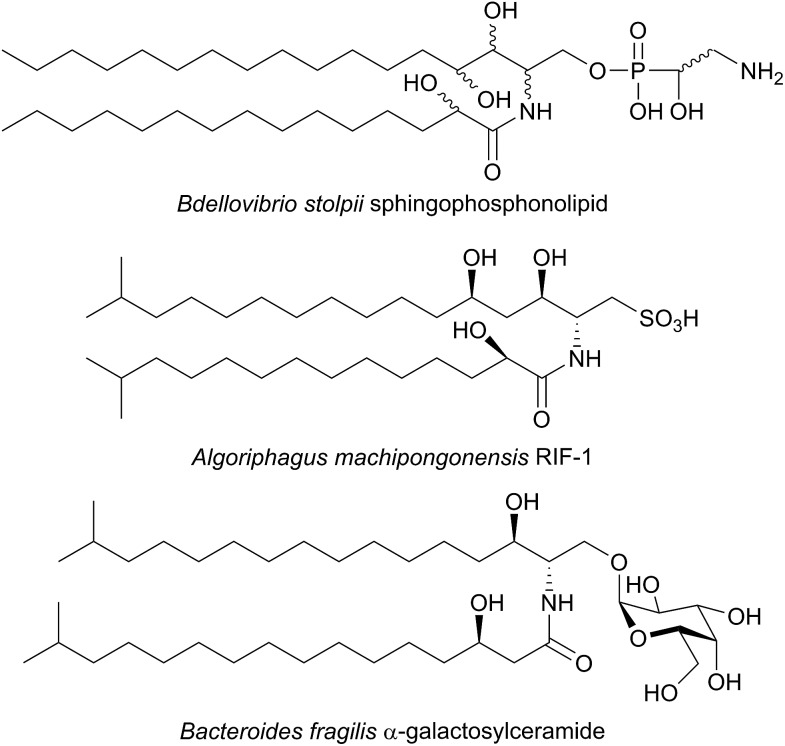
Structures of unusual bacterial sphingolipids. *Bdellovibrio stolpii* sphingophosphonolipid (stereochemistry not reported), RIF-1 from *Algoriphagus machipongonensis* and *Bacteroides fragilis* isobranched galactosylceramide.

SPT has been identified in other *Sphingomonas* bacterial strains including *S. multivorum* (A7BFV6), *S. spiritivorum* (A7BFV7) *S. wittichii* (A5VD79) and *Bdellovibrio stolpii* (*Bacteriovorax stolpii*, A7BFV8) with each enzyme displaying high sequence homology and biochemical properties similar to the *S. paucimobilis* SPT.^42^,^54^ *B. stolpii* is an interesting case in that it contains a unique SL head group with an unusual phosphonate linkage to the C1 of the sphingoid base, which appears to be important in the lifecycle of the organism (Fig. 4).^55^,^56^ There is some evidence that, *in vivo*, the SPTs from *S. multivorum* and *B. stolpii* SPT cluster around the inner cell membrane suggesting that these SPT enzymes may release the highly hydrophobic KDS product directly into the membrane. Interestingly, in the *S. wittichii* genome, the SPT is located adjacent to an acyl-carrier protein (ACP).^54^ This has led to the hypothesis that *S. wittichii* SPT is able to accept C_16_ units from an ACP thioester rather than an acyl-CoA.

**Fig. 5:**
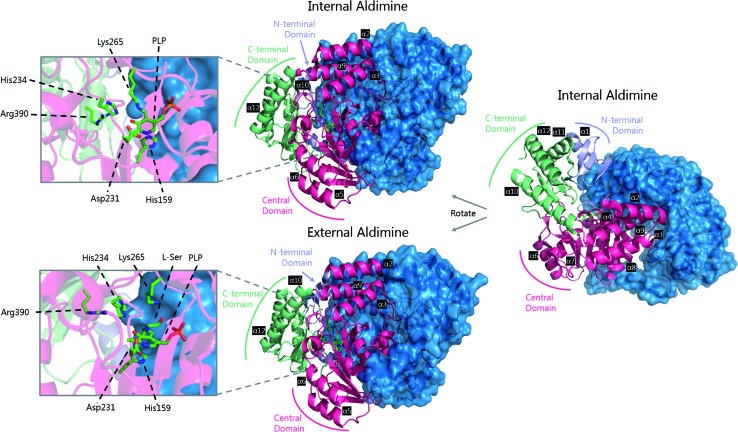
The 3D structures of *S. paucimobilis* SPT in the internal aldimine (top, PDB: ; 2JG2) and external aldimine (bottom, PDB: ; 2W8J) forms. The active sites are shown, highlighting key residues. To highlight the dimeric nature of SPT, one of the SPT monomers is shown in ribbon form, whilst the other is shown as a surface representation. An overlay of the active sites in the internal and external aldimine forms is shown in Fig. 6.

A crystal structure of *S. multivorum* SPT with bound PLP was first reported in 2006.^57^ However, the first published SPT structure with an associated Protein Databank (PDB) file detailing the X-ray coordinates (*S. paucimobilis* SPT, SpSPT) was determined by Yard *et al.* and reported in 2007 39 (Fig. 5). This structure was then followed by reports of the *S. multivorum* SPT^58^ (PDB: ; 3A2B) and *S. witichii* SPT (PDB: ; 2X8U).^54^ For clarity, here we will use the *S. paucimobilis* SPT sequence when referring to amino acid residues. Analysis of the structures of *S. paucimobilis* SPT reveals that SPT is a homodimeric complex, with an overall topology which resembles other type-1 PLP family enzymes such as AONS, KBL and ALAS.^39^ Each SPT monomer consists of three domains; N-terminal, central catalytic and C-terminal, all three of which are involved in the dimerization of SPT. The N-terminal domain is short, comprising only 80 amino acids which form an α-helix and an antiparallel β-sheet. The central catalytic domain is dominated by a seven stranded β-sheet structure, which is characteristic of type-1 PLP enzymes. The catalytic lysine, Lys265, is located in this domain. Finally, the mostly α-helical, C-terminal region consists of approximately 100 residues. The SPT active site is formed at the dimer interface and contains the active site lysine to which the PLP attaches as the internal aldimine.^39^ Residues from both subunits form the active site, and since bacterial SPT is a homodimeric complex there is a 1 : 1 stoichiometry between the number of subunits and the number of active sites (*i.e.* two active sites per dimer). The two long hydrophobic channels that lead from the active site to the surface of the enzyme have been proposed to act as binding pockets for the hydrophobic palmitoyl-CoA substrate.^39^,^58^ However, no electron density corresponding to palmitoyl-CoA has been found in crystals of SPT, therefore which channel is required for palmitoyl-CoA binding is unknown.

The determination of the X-ray structure of the SpSPT PLP:l-serine complex by Raman *et al.* (PDB: ; 2W8J), allowed a comparison of the internal (PLP-bound) and external (PLP:l-serine) aldimine forms of the enzyme.^59^ There is an extensive hydrogen bonding network between the PLP cofactor and residues of the active site. Of particular note is His234, which hydrogen bonds with the PLP hydroxyl and Asp231 which hydrogen bonds with the protonated pyridine nitrogen (protonation of the pyridine nitrogen is required for α-proton abstraction in PLP enzymes).^60^ The phosphate of PLP forms the centre of a phosphate binding cup, hydrogen bonding with residues of both subunits.^39^,^41^ These interactions are all retained in the external aldimine form of the enzyme.^58^,^59^

**Fig. 6:**
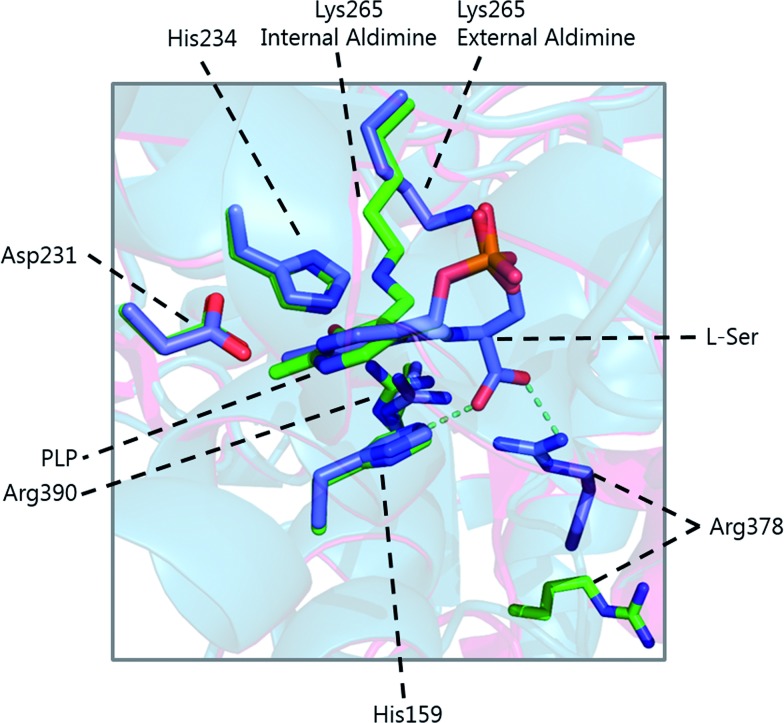
Overlay of the internal (green carbon atoms) and external (purple carbon atoms) of *S. paucimobilis* SPT. Hydrogen bonds of l-serine to His159 and Arg378 are also shown. The large movement of Arg378 from the internal to external aldimine forms is evident.

Formation of the SPT:PLP–l-serine external aldimine complex is accompanied by several structural changes (Fig. 6). His159 sits on one face of the PLP-pyridine ring, forming a π–π stacking interaction and is required for PLP binding to the enzyme. This histidine residue is the first of three consecutive residues (His–Ala–Ser) which are strictly conserved amongst AOS family members. Mutational analysis suggests that His159 plays multiple roles in SPT with involvement in substrate recognition and catalysis.^61^ It is proposed that His159 aids in recognition of l-serine by forming a hydrogen bond with the carboxylate of the l-serine. This is important as it ensures that the Cα-COO^–^ bond is almost perpendicular to the plane of the pyridoxal nitrogen of the PLP–l-serine-external aldimine.^62^ As suggested by Dunathan, it is the bond perpendicular to the plane of the pyridoxal nitrogen which is preferentially cleaved.^63^ By fixing the conformation of the external aldimine, deprotonation is prevented until binding of the second substrate, palmitoyl-CoA, thus preventing the enzyme from undergoing an abortive transamination reaction. Binding of palmitoyl-CoA is proposed to disrupt the hydrogen bond of His159 with the l-serine carboxylate, which results in rotation of l-serine, allowing the carboxylate to form an interaction with Arg390. These structural changes position the Cα–H bond of l-serine perpendicular to the plane of the pyridine nitrogen, allowing deprotonation to occur and the reaction to proceed. The role of Arg390 in catalysis after the formation of the external aldimine is supported by mutational experiments.^40^ In *S. witichii*, when the arginine analogous to Arg390 (Arg370) is mutated, formation of the external aldimine is not perturbed.^54^ However, when *S*-(2-oxoheptadecyl)-CoA is added to the reaction, no substrate quinonoid is formed (that can be detected).

Other changes also occur on the formation of the external aldimine. Of note is the positioning of Lys265, which moves approximately 4 Å. This results in a hydrogen bond between Lys265 and the hydroxyl group of l-serine.^58^,^59^ The hydroxyl of l-serine hydrogen bonds with the PLP phosphate, contributing to the phosphate binding cup.^41^ This interaction has been shown to be required for optimal activity of the SPT enzyme. Additionally, in the external aldimine form, Arg378 swings into the active site, forming a salt-bridge with the carboxylate of the l-serine on the external aldimine.^58^,^59^ The effect of these changes in the structure on the formation of the external aldimine is to ensure, as discussed above, that the Cα–H bond is not perpendicular to the pyridoxal nitrogen.

A variety of different SL-like natural products have been identified in bacteria. The marine bacterium *Algoriphagus machipongonensis* co-habits in the same environment with the marine organism *Salpingoeca rosetta* and produces a signalling compound known as Rosette Inducing Factor (RIF, Fig. 4).^64^ Although chemically similar to an SL, RIF is in fact a sulfonolipid which is required to trigger multicellular rosette colony formation in *S. rosetta*. Similarly, SL-like natural product inhibitors of SPT have been identified in bacteria and fungi (see Section 2.8). Although the precise biosynthetic pathways of these compounds is unknown, retro-biosynthetic analysis suggests that an SPT-like reaction could be involved. If true, this demonstrates the central role that SPT-like enzymes and other AOSs play in the biosynthesis of many different compounds.

Additional interest in bacterial SPTs has been generated through the investigation of the roles that SLs play in *Bacteroides*, the most abundant genus of human gut commensal bacteria.^65^ Of particular interest is *Bacteroides fragilis*, an opportunistic pathogen which has been linked to inflammatory bowel disease and colon cancer and whose membrane consists of 40–70% SLs.^66^,^67^ *B. fragilis* has been suggested to modulate the host immune system through α-galactosylceramides, which are proposed to be produced from iso-branched sphingoid bases (Fig. 4).^68^,^69^ Consequentially, an SPT-like enzyme probably catalyses a key step in the biosynthesis of these molecules. A putative SPT with high homology (32.5% identity) to SpSPT has recently been identified in the oral dental pathogen *Porphyromonas gingivalis* (a *Bacteroides*),^70^ a bacteria known to produce sphingolipids.^71^–^73^ Deletion of the gene (PG1780) encoding this enzyme from strain W83 abolished SL production, diminished the long-term survival of *P. gingivalis* and impacted the cell surface properties of the PG1780 mutant strain. A detailed understanding of the biosynthesis of SLs in the human microbiome (both commensals and pathogens) will shed light on their roles in mediating host–bacteria interactions, as well as potentially aiding in the development of novel antimicrobial therapeutics. The role of sphingolipids in host–microbial interactions has recently been eloquently reviewed,^74^ and sphingolipids have been identified in other oral microbiome bacterium by Nichols.^75^

### 2.3 SPT in viruses

Large scale genomic sequencing has allowed the identification of SPT genes in different organisms from interesting and unexpected biological niches. Genes encoding SPT and other proteins involved in SL metabolism have been found in the marine virus *Coccolithovirus*, a pathogen which infects the plankton *Emiliania huxleyi*.^76^ In a seminal study, it was shown that viral SLs induce lysis and cell death which leads to the breakdown of algal blooms.^77^–^79^ These viral-derived glycosphingolipids can cause programmed cell death in an uninfected *E. huxleyi* host and were detected in high enough concentrations in the coccolithophore populations in the North Atlantic Ocean to be deemed effective biomarkers for viral infection. Interestingly, the SPT gene in *Coccolithovirus* is a natural chimera of two subunits, analogous to the eukaryotic SPT subunits (SPT1 and SPT2, see Section 2.5 below), and is expressed as a single polypeptide chain.^76^,^80^ The N-terminal domain of the protein is predicted to contain the essential, conserved lysine residue required for binding of the PLP cofactor (making it analogous to SPT2), whilst the C-terminal domain is, by sequence alignment, analogous to SPT1.^80^ The *Coccolithoviral* SPT chimera was able to complement growth in yeast lacking endogenous SPT, although the activity of *Coccolithoviral* SPT was significantly lower than the SPT from yeast. Significantly, *in vitro* assays using microsomes containing the *Coccolithoviral* SPT revealed a preference for C_14_-CoA (myristoyl-CoA) as a substrate over C_16_-CoA (palmitoyl-CoA). Infection of *E. huxleyi* with *Coccolithovirus* also alters the LCB profile of the host, ‘rewiring’ ceramide synthesis in *E. huxleyi*.^81^ A recent biochemical study of the *Coccolithovirus* and *E. huxleyi* pathways suggests that the viral SPT encodes the key switch that diverts metabolism towards the formation of the toxic SL which ultimately leads to the death of the phytoplankton.^81^

### 2.4 SPT in yeast and fungi

**Fig. 7:**
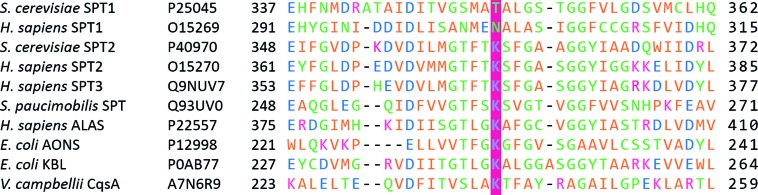
Partial sequence alignment of serine palmitoyltransferases with other AOS family members (ALAS, CqsA, KBL and BioF), highlighting the conserved lysine residue which is not present in the SPT1 subunits of SPT heterodimers.

Over many years the yeast *S. cerevisiae* has played a pivotal role in defining the genes, pathways and enzymology of SL biosynthesis in a higher eukaryote. In yeast and other higher order species, SL biosynthesis occurs in the ER. SPT was identified in yeast through genetic screens, one designed to find auxotrophs that required LCBs for growth^82^–^86^ and another that identified suppressors of the csg2Δ mutant, defective in complex sphingolipid biosynthesis.^87^ It was found that two genes, *lcb1* (long chain base) and *lcb2* were required for SPT activity. Both encoded gene products, LCB1 and LCB2, belonging to the AOS family of enzymes, and deletion of either *lcb1* or *lcb2* was found to abolish SL biosynthesis in yeast.^85^,^87^ For the sake of simplicity, we will refer to LCB1 as SPT1 and LCB2 as SPT2. Sequence alignment of SPT1 and SPT2 with other members of the AOS family shows that whilst SPT2 contains the conserved lysine required for PLP binding, SPT1 does not (Fig. 7).^82^,^88^,^89^ In view of this observation, an early hypothesis was that SPT2 was a “catalytic” subunit and SPT1 performed some regulatory role in the SPT-catalysed reaction. Modelling studies with AONS led to the suggestion that the SPT active site lay at the interface between the two subunits of an SPT1/SPT2 heterodimer.^90^ Subsequently, determination of the bacterial SpSPT structure and mechanism highlighted a number of residues required for l-serine and acyl-CoA substrate binding and catalysis; namely His159, His234, Asp231, Arg390 (numbering in SpSPT). Residues analogues to these amino acids are lacking in SPT1.^39^,^40^,^61^

Immunoprecipitation experiments demonstrated that SPT1 and SPT2 form a heterodimeric complex, presumably containing a single active site, with the PLP binding lysine provided by SPT2.^82^,^90^ This heterodimeric arrangement is unique amongst AOS family members since others are homodimers. However, this raises questions as to the role of SPT1, and whether it has a regulatory function (see Section 2.7). Further experiments indicated that SPT2 is unstable in the absence of SPT1, suggesting a more intimate dimeric partnership.^91^ However, the PLP binding capacity of either SPT1 or SPT2 has yet to be demonstrated *in vitro*. Topological experiments revealed that SPT1 consists of three transmembrane helices with the N-terminal domain located in the ER lumen and the C-terminal domain located in the cytosol.^92^ Moreover, the central and C-terminal domains of the enzyme were shown to be required for the stability of the SPT2 subunit through site directed mutagenesis and deletion experiments. These data, taken together, suggest that SPT1 and SPT2 interact in both the membrane and in the cytosol and that in the absence of SPT1, SPT2 is not stable.

An important third SPT subunit was identified by Dunn and co-workers, termed Tsc3p.^91^ Tsc3p was shown to be a novel 80 amino acid membrane-associated protein which co-immunoprecipitates with SPT1 and SPT2, but is not essential for SPT activity. However, microsomal preparations from a tsc3Δ *S. cerevisiae* mutant were shown to have 30-fold lower SPT activity than those from the wild type strain, showing that Tsc3p is required for optimal, high level SPT activity during maximal SL biosynthesis. The exact molecular details of how Tsc3p exerts its stimulating activity on the SPT complex is unknown.

### 2.5 SPT in mammals

Once the genes that encode SPT in yeast had been discovered it was not long before the homologues of yeast SPT1 and SPT2 were identified in humans (SPT1: O15269 and SPT2: O15270), mice (SPT1: O35704 and SPT2: P97363) and other mammals *via* sequence analysis and functional complementation assays.^93^–^95^ Between human and yeast SPT1 there is 30.9% identity and 46.3% similarity whilst between human and yeast SPT2 there is 42.2% identity and 60.7% similarity, suggesting that the roles of SPT1 and SPT2 in mammals and yeast are analogous.^95^ As with the yeast SPT1 and SPT2 isoforms, experiments with the mammalian SPT1 and SPT2 indicted that both were absolutely required for catalytic activity, and mutation of the putative PLP cofactor binding lysine residue in SPT2 abolished catalytic activity even in the presence of SPT1, which lacks the PLP binding motif.^93^ Additionally, levels of mRNA transcripts for the SPT1 and SPT2 subunits of SPT correlate in their tissue distribution, as is to be expected for subunits of the same complex.^95^

Detailed biochemical characterisation of the SPT complex was not achieved until 2000 when the SPT complex from hamster was purified by Hanada and co-workers *via* the use of affinity peptide chromatography.^96^ They took advantage of a LY-B strain, which is a CHO cell line defective in SPT activity due to a G246R mutation in the SPT1 protein.^97^ Purified SPT was found to show a strong preference for l-serine. The acyl-CoA specificity of the SPT complex was also found to be quite narrow. Although palmitoyl-CoA (C_16_) was the preferred substrate, other acyl-CoA substrates, such as pentadecanoyl- (C_15_) and heptadecanoyl-CoA (C_17_) were also accepted. Shorter (myristoyl-CoA, C_14_), longer (stearoyl-CoA, C_18_) and modified acyl-CoA substrates (arachidonoyl- and palmitoleoyl-CoA) were not turned over. Using a combined western blot/radiolabelling/immunoprecipitation approach, the stoichiometry of SPT1 and SPT2 in the mammalian complex was determined to be 1 : 1, suggesting a heterodimeric complex, although a heterotetrameric complex is also possible.^96^ Interestingly, the nature of the SPT1 defect in the useful LY-B CHO cell line was not resolved until 2009 by Merrill, Hanada and colleagues. By sequencing the SPT1 transcript from LY-B cells they found a single mutation (G246R) which caused the translated protein to be unstable and thus barely detectable in protein and SPT assays. They also attempted to model why such a mutation would be so deleterious to the SPT1 protein and predicted this residue could not be accommodated in a conserved hydrophobic pocket.^97^

**Fig. 8:**
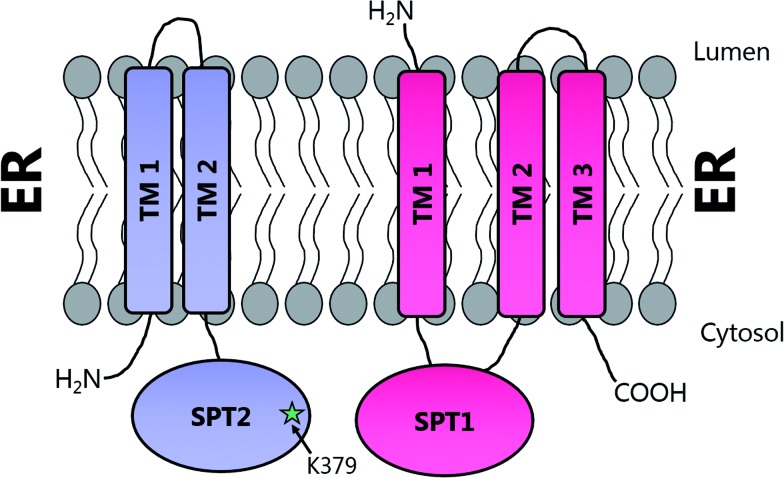
Proposed transmembrane domain topologies of human SPT1 and SPT2 within the ER membrane. The active site lysine required for PLP binding is located on SPT2.

Topological studies on mammalian SPT1 give results that are consistent with those obtained on yeast SPT1 (Fig. 8).^92^,^98^ Mammalian SPT1 is localised to the ER with the N-terminus of the protein in the ER lumen and the C-terminus exposed to the cytosol. Similar to its yeast homologue, human SPT1 was also found to be necessary for the stability of SPT2. The positioning of the termini of SPT1 indicates that SPT1 must have an odd number of transmembrane helices. Yasuda *et al.* suggest that human SPT1 contains a single transmembrane spanning domain, since other domains in SPT1 have similarity to soluble AOS family members and that other hydrophobic regions form the internal region of a large soluble globular domain. Subsequent work by Han *et al.* on yeast SPT1 cast doubt on this initial topology model. By inserting the glycosylation cassette from the invertase Suc2p at various sites into SPT1 and monitoring whether the resultant protein was glycosylated or not, Han and co-workers obtained results which led them to propose that SPT1 has three transmembrane domains. This model is further supported by protease accessibility studies. These topological studies remain the only structural insight into the human SPT complex since there is, as yet, no high resolution data for mammalian SPT1 or SPT2.^92^

Detailed understanding of the human SPT complex has been further complicated by the identification of a second homologue of yeast SPT2 in humans, termed SPT3 (SPTLC3), or SPT2b (Q9NUV7). SPT3 shows 68% homology to SPT2, has similar enzymatic functions and is proposed to form a dimer with SPT1 in a fashion similar to SPT2.^99^ However, the tissue expression pattern of SPT2 and SPT3 vary, with the highest expression of SPT3 in both placenta and human trophoblast suggesting that it may alter either the activity of the SPT complex or the substrate specificity of the complex.^99^ This turned out to be correct when it was found that the acyl-CoA chain selectivity of a SPT complex containing SPT3 displayed a preference for C_14_-CoAs and thus generated C_16_-derived SLs in the tissues expressing this subunit.^100^

Additionally, motivated by their discovery of Tsc3p and the observation that co-overexpression of SPT1 and SPT2 did not cause a corresponding increase in SPT activity in mammalian cells, Dunn and colleagues identified small subunits of human SPT, functionally analogous to Tsc3p in yeast, termed ssSPTa (small subunit) and ssSPTb.^101^ These human SPT subunits are small (9 kDa), hydrophobic proteins that display 57% sequence similarity and 36.7% identity to each other. However, neither ssSPTa nor ssSPTb displays significant sequence homology to Tsc3p. As such, ssSPTa/b were identified using an spt1Δspt2Δ *S. cerevisiae* double knockout strain (TDY8055) in which expression of the human SPT1/2 without a small subunit supports growth at 26 °C. Growth is permitted at 37 °C only by the addition of the yeast-specific SL phytosphingosine. In order to identify the human small subunits, a human cDNA library was transformed into the yeast strain expressing human SPT1 and SPT2 and transformants able to grow at 37 °C were recovered. Two functional orthologues of Tsc3p were identified, ssSPTa and ssSPTb. The *ssSPTb* gene had been previously identified in a screen for genes that were down regulated by androgen in mouse prostate, but no function had been assigned, and since the small subunits were found to co-purify with SPT and SPT2a/b they were assigned as *bone-fide* SPT subunits. Moreover, when ssSPTa was co-expressed with SPT1 and SPT2 in microsomal preparations of LY-B CHO cells, SPT activity was increased over 100 fold. Most interestingly, the resultant four different possible isoforms of the SPT complex (SPT1–SPT2a–ssSPTa, SPT1–SPT2a–ssSPTb, SPT1–SPT2b–ssSPTa, SPT1–SPT2b–ssSPTb) have distinct acyl-CoA specificities. The SPT1–SPT2a–ssSPTa isoform conferred a strong preference for C_16_-CoA, whilst the SPT1–SPT2b–ssSPTa isoform was equally accepting of C_14_-CoA as C_16_-CoA. In contrast to SPTs containing the ssSPTa isoforms, those with the ssSPTb subunit display a preference for longer acyl-CoA substrates, up to C_20_-CoA. For all four isoforms, the *K*_M_ for l-serine (∼1–2 mM) was the same with the preferred acyl-CoA substrate. The acyl-CoA preference of SPT complex can also be directly influenced by mutation of a single residue in the small subunits, demonstrating that the small subunits are crucial for controlling the acyl-CoA preference of SPT.^102^,^103^ A more recent study has identified a H65K mutation in ssSPTb in mice, referred to as the *Stellar* mutation.^102^ The effect of this mutation is to increase the proportion of C_20_ containing long chain bases in tissues, in particular the eyes and brain, leading to a “eye flecking” phenotype in young mice. Neuronal damage is also observed, including early onset ataxia and death at 10 weeks in homozygous mice carrying this single mutation. In microsomal assays, SPT activity was unaffected with a C_16_-CoA substrate but doubled with C_18_-CoA. The exact molecular details of how elevated SLs in the Stellar mice lead to neuropathy and increased mortality are unclear.

Deletion experiments have shown that with the small subunit SPTs, neither the N- nor C-terminal domains are essential.^103^ It is only the core 33 amino acids of ssSPTa which are required for activation of the SPT heterodimer and for conferring the acyl-CoA specificity of the complex. Of interest, a single residue, Met25 in ssSPTa and Val25 in ssSPTb dictates the acyl-CoA specificity. Surprisingly, a single M25G mutation in ssSPTa results in an alteration of the acyl-CoA specificity towards longer (ssSPTb-specific C_20_) acyl-CoA substrates suggesting this residue confers substrate specificity. Topological analysis demonstrate that the N-termini of both ssSPTs is cytoplasmic and suggest that the ssSPTs have a single transmembrane spanning domain, which comprises the core 33 amino acid region.^101^,^103^ However, at the molecular level, it is unknown how the small subunit is able to confer these changes in substrate specificity and how it interacts with the larger SPT complex. The N-terminal domain of SPT1 can be deleted and the resulting truncated form still displays enhanced SPT activity in the presence of SPT2 and either of the small subunits,^92^ indicating this part of SPT1 does not interact with its small activating partners. That said, it is also worth noting that deletion of the N-terminal transmembrane domain of SPT1 does not prevent membrane association or SPT activity, clearly supporting the presence of additional membrane spanning domains.

The observation of acyl-CoA substrate promiscuity in the eukaryotic SPT isoforms suggests that diversification in the types of SLs produced begins very early in the SL biosynthesis pathway, leading to a sphingolipidome pool of large structural variation.^7^ This also raises questions as to how downstream enzymes after SPT are able to cope with the broad substrate range, and how the biosynthesis of different types of SLs is controlled.

### 2.6 Sphingolipids and disease

Sphingolipids have been linked to a number of human diseases and pathologies. These include Alzheimer's, cancer, diabetes and various inflammatory diseases.^5^ The molecular mechanisms of these diseases and how they relate to sphingolipid homeostasis and any disease-driven imbalance, is the subject of current intensive studies.^104^,^105^ The genetic susceptibility to a particular disease and the role that an individual's sphingolipid “inventory” (determined by their specific biosynthetic pathway) is also under investigation. An ideal scenario would be a clear link between a specific gene mutation, an aberrant enzyme activity and a resultant disease phenotype. However, such clear cut links are rarely observed. Here we highlight the current work on a rare neuropathy where mutations in SPT provide a direct link between mutation, enzyme activity and disease.

#### 2.6.1 Deoxysphingolipids and the link to HSAN1

**Fig. 9:**
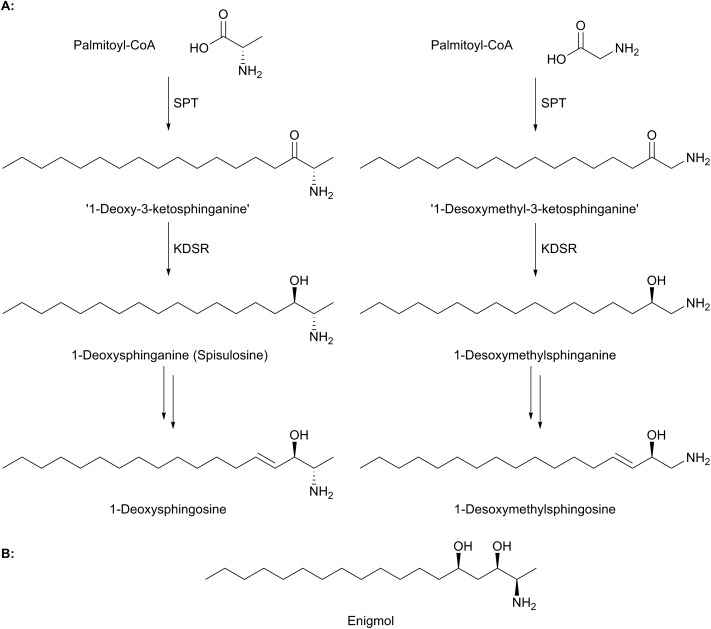
(A) Structures of deoxy-SLs formed from the condensation of palmitoyl-CoA with l-alanine (left) and glycine (right) forming ‘1-deoxy-3-ketosphinganine’ from l-alanine and ‘1-desoxymethyl-3-ketosphingaine’ from glycine. These are reduced to 1-deoxysphinganine and 1-desoxymethylsphinganine respectively and then further metabolised to 1-deoxysphingosine and 1-desoxymethylsphingosine. It should be noted that addition of the double bonds at C4 and C14 probably occurs to the *N*-acyl-deoxysphingoid base. (B) Structure of the natural product deoxyl-SL enigmol.

Mutations in the genes encoding SPT have been associated with the rare disease hereditary sensory and autonomic neuropathy type 1 (HSAN1).^17^ HSAN1 is an autosomal dominant disease that results in progressive distal sensory loss and ulceration of the limbs resulting from degeneration of dorsal root ganglia and motor neurons.^18^,^106^,^107^ The link between HSAN1 pathology and SPT has been hypothesised to be a consequence of a gain-of-function of HSAN1 SPT mutants that can condense glycine or l-alanine with acyl-CoAs^108^,^109^ (Fig. 9) to form ‘1-deoxy-3-ketosphinganine’ or ‘1-desoxymethyl-3-ketosphinganine’ (this intermediate is only observed when ceramide synthase is inhibited with fumonisin since deoxy-SLs are predominantly present in the *N*-acylated form^19^). The lack of a C1 hydroxyl on LCBs derived from these amino acids prevents the resulting so-called “deoxy-SLs” from being phosphorylated by LCB kinases (1-deoxysphinganine and 1-deoxysphingosine from l-alanine and 1-desoxysphinganine and 1-desoxysphingosine from glycine). Phosphorylation of LCBs is required for the action of the lyase enzyme responsible for the degradation of LCBs (see Section 5.0). As such, the resulting deoxy-SLs cannot be degraded by the canonical degradation pathway and accumulate in membranes, resulting in an ER stress response and cellular toxicity.^108^–^111^ There is evidence, however, that deoxy-SLs are metabolised by a cytochrome P450 dependent pathway, which results in hydroxylation and dehydration of the C14–C15 bond.^112^ Hornemann and colleagues suggest this may be a deoxy-SL degradation pathway (degradation products beyond these hydroxylation and desaturation reactions are unknown) and that a link between patients with type 2 diabetes and increased deoxy-SLs may be due to downregulation of these enzymes.^112^

Evidence suggests that wild-type, functional SPT can form 1-deoxy-LCBs under certain stress conditions such as impaired glucose, lipid or amino acid homeostasis.^113^ That nature already produces these “toxic” molecules is well known and has been elegantly reviewed by Merrill.^19^ Briefly, there are a number of deoxys-SL natural products that have attracted great interest as lead molecules in anti cancer therapy. For example, the clam-derived compound spisulosine (Fig. 9a) is an inhibitor of cell proliferation and triggers cell death pathways in several cancer cell lines, with an anti-proliferative IC_50_ of 1 μM in prostate tumour PC-3 and LNCaP cell lines.^114^–^116^ Similarly, enigmol (Fig. 9b) is another sphingolipid analogue which can be administered orally to mice and is toxic to a number of different cell lines including colon cancer, and in mouse xenographs with prostate cancer.^117^

#### 2.6.2 HSAN1 mutations in SPT1

To date 15 HSAN1-causing SPT mutations (on both SPT1 and SPT2) have been identified in patients from the USA, Europe and Australia (Table 2).^118^ HSAN1 patients display a wide range in age of onset of symptoms, disease severity and clinical outcome. Historically, one of the first mutations found to cause HSAN1 was mapped in 2001 to the SPT1 gene (located on chromosome 9) of HSAN1 patients in both American and Australian families.^17^,^18^

**Table 2:** Summary of HSAN1 causing mutations in the SPT1 and SPT2 subunits of SPT

LCB subunit	Mutation	Clinical effect	Biochemical effect	Ref.
LCB1	C133W	Sensory neuropathy, ulceromutilations, lancinating pains	Increased activity towards l-alanine (*K*_M_ 9.6 mM), activity towards l-serine unaffected (*K*_M_ 0.75 mM for WT, 1.4 mM for mutant)	18, 109 and 111
LCB1	C133Y	Sensory neuropathy, ulceromutilations, lancinating pains	Increased levels of deoxysphingoid bases. Reduced SPT activity	18, 109 and 111
LCB1	C133R	Sensory neuropathy	Increased levels of deoxysphingoid bases. Reduced SPT activity	118
LCB1	V144D	Sensory neuropathy, ulceromutilations, lancinating pains	Decreased SPT activity	90, 109 and 110
LCB1	A310G	Uncertain pathological significance	Unknown	123
LCB1	S331F	Hypotonia, ulcerations, severe growth and mental disability, vocal cord paralysis, gastroesophageal reflux	Increased levels of deoxysphingoid bases. Reduced SPT activity	120–122
LCB1	S331Y	As S133F	Increased levels of deoxysphingoid bases. Reduced SPT activity	120–122
LCB1	A352V	Sensory neuropathy, ulceromutilations, lancinating pains	Reduced SPT activity. Does not lead to increased deoxysphingoid base levels *in vitro*	120 and 121
LCB1	G387A	Not disease associated	Not disease associated	122
LCB2	A182P	Reduced sensation in the feet, sensory loss and motor weakness	Increased levels of deoxysphingoid bases. Reduced SPT activity. Increased activity *vs.* alanine	124
LCB2	R183W	Mild late onset progressive distal sensory impairment	Elevated 1-deoxysphingolipid levels. No effect on SPT activity	127
LCB2	V359M	Sensory neuropathy, ulceromutilations	Ambiguity towards amino acid substrate	126
LCB2	G382V	Sensory neuropathy, ulceromutilations	Ambiguity towards amino acid substrate	126
LCB2	S384F	Reduced sensation in feet, shooting pains, ulcerations and motor weakness	Reduced activity to l-serine, increased activity to l-alanine	113
LCB2	I504F	Sensory neuropathy, ulceromutilations, osteomyelitis and anhidrosis	Ambiguity towards amino acid substrate	126

The most common mutation was discovered to be due to a single missense mutation of Cys133 to either a tryptophan or a tyrosine (C133W or C133Y).^18^ Modelling studies based on the structure of bacterial SPT suggested that Cys133 is located in close proximity to the active site of the SPT1–SPT2 heterodimer, across the subunit interface from the SPT2 lysine required for PLP binding,^39^,^59^,^90^ and initial analysis suggested that pathology arose from reduced activity of the SPT heterodimer.^90^ In an important breakthrough, it was found that the HSAN1 mutations reduced promiscuous SPT activity leading to increased levels of toxic deoxysphingoid bases.^109^ In subsequent *in vitro* experiments expressing the human SPT1^C133W^–SPT2–ssSPTa/b heterotrimer in an *S. cerevisiae* spt1Δspt2Δ knockout, the mutant SPT enzyme was shown to have 10–20% activity of the wild type enzyme and in addition, produced C_18_-1-deoxysphingolipids (with ssSPTa and ssSPTb) and C_20_-1-deoxysphingolipids (with ssSPTb).^111^ When expressed in HEK293 cells, the SPT1^C133W^ mutant gene was shown to cause an increase in the levels of deoxysphingoid bases present.^109^ Hence the C133W mutation allows SPT to catalyse condensation with alanine, without altering the acyl-CoA specificity or significantly altering affinity for l-serine. SPT1^C133W^–SPT2a–ssSPTa has a *K*_M_ of 1.4 mM for serine and 9.6 mM for alanine, whereas the wild type enzyme has a *K*_M_ of 0.75 mM for l-serine and does not turnover alanine sufficiently to allow kinetic characterisation.^111^

**Fig. 10:**
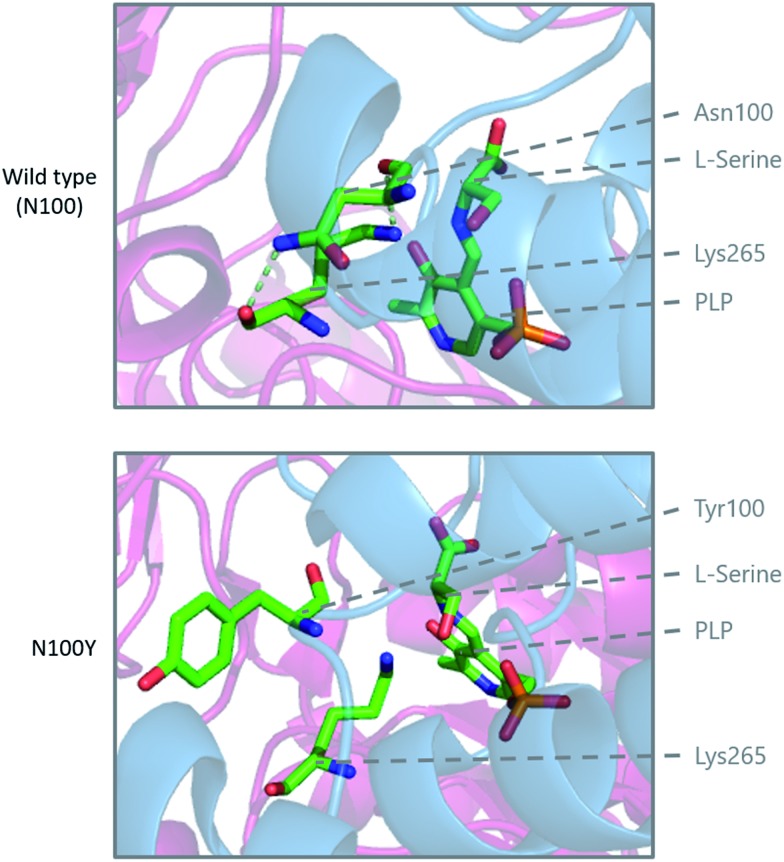
Modelling of the HSAN1 causing C133Y mutation of human SPT in *S. paucimobilis* SPT (N100Y, PDB: ; 2W8W), highlighting the structural changes which are proposed to affect the dimer interface and loss of hydrogen binding interactions in the active site.

Modelling of the C133W and C133Y mutations in *S. paucimobilis* SPT (where human SPT1 Cys133 corresponds to bacterial SPT Asn100) provides further insight into the structural effect of these mutations (Fig. 10). Analysis of purified so called “bacterial HSAN1 mutant mimics” (N100Y and N100W) caused a blue-shift of the ketoenamine peak of the PLP cofactor, indicating that the PLP binding environment is altered with respect to the wild type enzyme. For both mutants, l-serine substrate binding is diminished with respect to the wild type, as is the catalytic efficiency using C_16_-CoA.^59^ Structural analysis of the bacterial SPT N100Y mutant revealed that this mutant causes significant structural changes across the dimer interface, *i.e.* a mutation in one monomer impacted on the other, analogous to the human SPT1/SPT2 complex. So, it appears likely in the human enzyme that the structural impact of the HSAN1 mutants may be to perturb the passage of information between the monomers of the heterodimers.^59^ Interestingly one other more recently-identified mutation at Cys133 in SPT1 which leads to HSAN1 is C133R.^119^ The C133R phenotype was found to be mild with respect to other Cys133 mutations, indicating the biochemical features of this mutation may differ from that of C133Y and C133W.^119^

In a similar fashion to the Cys133 mutations, SPT1 V144D also decreases the enzymatic activity of the SPT1–SPT2 heterodimer in a yeast model.^90^ A similar effect on activity was observed when assayed in HEK293 cells overexpressing the mutant enzyme. This was coupled with an increase in the levels of deoxysphingoid bases in plasma.^109^,^110^ Val144 is predicted to be close to the enzyme active site,^39^,^59^,^90^ and so mutations here may affect the substrate binding capacity of the enzyme.

The most severe HSAN1 mutation identified is SPT1 S331F which results in hypotonia, ulcerations, severe skeletal growth defects and mental disability.^120^,^121^ Biochemically, decreased enzyme activity is observed for the SPT1 S133F and S133Y mutants in HEK293 cells, without a change in overall sphingoid base levels.^121^,^122^ In S331F/Y patients, elevated levels of 1-deoxysphingoid bases are found in patient's plasma.^121^,^122^ Molecular models using the bacterial SPT suggest that S331 is surface-exposed and so consequently mutation of S331 could impact on association with other proteins such as the small subunit or regulatory proteins. Interestingly, Dunn and colleagues identified the S331F mutation in SPT1 as responsible for elevated activity of the SPT1/SPT2 heterodimer. In addition, in contrast to the wild type heterodimer, the mutant heterodimer was activated better by ssSPTb than by ssSPTa.^103^ An SPT1 A352V mutation also generates a HSAN1 phenotype and increased levels of 1-deoxysphingoid bases in patients.^120^,^121^ Like Ser331, Arg352 is predicted to be surface exposed. However, *in vitro* analysis of the A352V mutation does not lead to an increase in levels of 1-deoxysphingoid bases.^120^,^121^ In contrast to other mutants, the SPT1 mutation, G387R, is said to be benign and has no clinical effect, nor does it result in decreased activity in HEK293 cells,^110^ whilst another, A310G, has been identified in an HSAN1 patient, but the pathological effect is uncertain, as is the effect on SPT activity.^123^

#### 2.6.3 HSAN1 mutations in SPT2

Of the 15 HSAN1 mutations identified so far, only 6 have been linked to the SPT2 subunit. Clinically, patients with an SPT2 A182P mutation present with reduced sensation in the feet, sensory loss and motor weakness.^124^*In vitro* characterisation of the A182P mutant showed 65% reduced activity compared to the wild type enzyme and increased activity towards alanine. Additionally, the mutant enzyme was shown to produce 1-deoxysphingoid bases *in vitro* and elevated levels of 1-deoxysphingoid bases were present in the plasma of patients.^124^ An HSAN1 mutation has been identified at S384 (S384F),^113^ which is believed to be one of two phosphorylation sites on SPT2 (in addition to Tyr387), as shown by Olsen *et al.*^125^ The Ser384 mutant shows decreased activity towards serine and increased activity to alanine compared to the wild-type. In order to mimic the effect of phosphorylation of the SPT2 subunit, Ernst and co-workers created two other mutants, S384D (constitutively phosphorylated) and S384A (non-phosphorylated). Analysis of these mutants showed that, like the S384F mutant, S384A had decreased activity with l-serine but increased activity with l-alanine, as well as increased levels of 1-deoxy SLs. The S384D mutant behaved similarly to the wild-type enzyme, suggesting that phosphorylation of Ser384 switches the substrate specificity of SPT from l-serine to l-alanine.

Finally, four other missense mutations have been identified in SPT2.^126^,^127^ Three mutations occur at strictly-conserved residues, V359M, R183W and G382V, and the fourth, I504F, is semi-conserved across different species. When expressed in HEK293 cells, V359M, G382V and I504F were found to result in decreased SPT activity and increased levels of deoxysphingoid bases.^126^ R183W on the other hand did not affect SPT activity, but did cause increased levels of deoxy-SLs both in the patient and *in vitro*.^127^*In vitro* expression of SPT1–SPT2^V359M/G382V/I504F^ssSPTa/b in yeast lacking endogenous SPT revealed that the mutations did result in changes to SPT activity.^106^ However, the effect was significantly less than observed for SPT1 C133W, C133Y and V144D mutants. Using *S. paucimobilis* SPT as a structural model, the effects of the V359M, G382V and I504F mutations were modelled *in silico* (human SPT2 Val359, Gly382 and Ile504 map to bacterial SPT V246, Gly268 and Gly385 respectively). The effects of these HSAN1 mutant mimics on bacterial SPT activity and structure are varied, suggesting the each mutant displays subtle differences in substrate binding and catalysis.^106^

The debilitating impact of the various HSAN1-causing mutations has driven clinicians, scientific researchers and affected families to consider some therapeutic intervention to if not cure, at least delay the onset of the disease. Since the evidence suggests that the HSAN1 mutant SPT complex is promiscuous and accepts l-alanine (and glycine) as well as l-serine, it was suggested that oral administration of the natural substrate l-serine could “compete out” this deleterious activity and reduce circulating deoxy-SL levels below a toxic threshold in various tissues. Initial results were promising enough to undertake an NIH-funded clinical trial of this dietary supplement in a small patient cohort (NCT01733407).^128^ If successful, this simple pharmaceutical intervention could prove to be a great example of personalised medicine. In a similar study with a single Finnish female patient with the mild R183W HSAN1 mutation in SPT2, dietary supplementation with l-serine resulted in a robust lowering of 1-deoxy-SL levels and no direct side effects.^129^

### 2.7 SPT in other organisms (plants, *C. elegans*, kinetoplastid parasites, apicomplexan parasites and *Drosophila*)

In this review we have chosen to concentrate on mammalian and microbial SL biosynthesis. However, it is worth highlighting that other model systems from various species have begun to bring out the similarities and differences in the genes, enzymes, cellular compartmentalisation and regulation across the various species.

In plants, just as in mammals and yeast, SLs are important components of cell membranes, comprising up to 10% of plant lipids.^130^ Plant SLs have been shown to be important for a host of cell signalling pathways,^131^ such as stomata opening,^132^ low temperature signalling^133^ and hypoxia response.^134^,^135^ A more detailed overview of plant SL biosynthesis is provided in recent reviews by Markham *et al.*^8^ and Michaelson *et al.*^136^

**Fig. 11:**
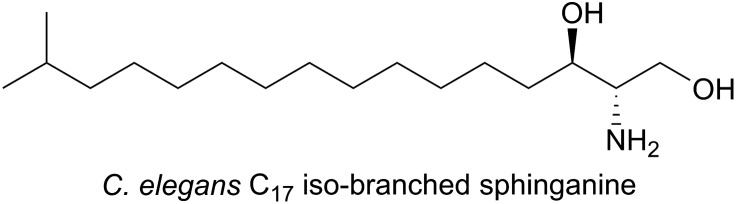
Structure of the C_17_ iso-branched sphinganine from *C. elegans*.

In the nematode worm *Caenorhabditis elegans,* SLs have been found to be important in various cellular functions especially in maintaining healthy and active mitochondria.^137^ In an elegant study, RNA interference (RNAi), combined with drug-induced or genetic disruption of mitochondria, led to the identification of 45 genes that were required to upregulate detoxification, relay a pathogen response and manage mitochondrial repair pathways. Surprisingly, the screen identified *C. elegans* SPT and other SL biosynthetic enzymes as playing roles in the surveillance of mitochondrial damage. The expression of the *C. elegans* SPT1-encoding gene was upregulated 2.5-fold by mitochondrial damage suggesting that increased sphingolipid levels may acts as a warning signal to the cell during mitochondrial disruption. *C. elegans* live in a bacterial-rich niche and some microbes antagonise mitochondria. In a more recent study, the unusual iso-branched (iso-methyl C_15_ and C_17_, Fig. 11) structures of the sphingoid bases from *C. elegans* were determined.^138^ These iso-branched sphingoid bases did not support yeast mutant cells lacking the ability to synthesise endogenous SLs. In *C. elegans* RNAi mutants lacking SPT1, disruption to intestinal function was observed, and only the natural iso-branched SLs, not deoxy-iso-branched SLs, could rescue this phenotype.

A recent study also investigated the SPT in the apicomplexan parasite *Toxoplasma gondii*. This protozoan parasite has been known to cause foetal damage and abortion in animals and humans. This organism can synthesize SLs *de novo* and also scavenge them from their host. For *de novo* synthesis, *T. gondii* has evolved two highly-homologous SPTs (TgSPT1 and TgSPT2).^139^ Bioinformatic analysis suggests that TgSPT1 and TgSPT2 evolved *via* a gene duplication event (a single copy is conserved in other Apicomplexa) after horizontal gene transfer from a prokaryotic species. Sequence analysis, combined with biochemical assays on the recombinant SPTs revealed that TgSPT1 is an active homodimer that displays the strongest similarity to the microbial SPTs. The TgSPT is ER-bound but the authors generated soluble protein by N-terminal truncation, thereby increasing the probability of being able to determine the structure of this unusual isoform, which, despite coming from a eukaryotic parasite, bears the strongest resemblance to the bacterial SPTs.

SPT activity has been identified the kinetoplastid parasite *Leishmania*.^140^ Denny *et al.* showed that *Leishmania major* contains homologs of both SPT1 and SPT2, similar to eukaryotes. LmSPT2 is resident in the ER and essential for sphingolipid biosynthesis (LmSPT2Δ mutants were severely growth compromised). Interestingly, LmSPT2Δ mutants are unable to differentiate into the infective forms of the parasite, suggesting a role in infectivity.^141^ Moreover, analysis of inositol phosphorylceramides using tandem mass spectrometry from *L. major* indicates that the preferred substrate for LmSPT is myristoyl-CoA. In contrast, the preferred substrate eukaryotes and the kinetoplastid parasite *Trypanosoma brucei* is palmitoyl-CoA.^142^–^144^

Similarly, the fruit-fly *D. melanogaster* has also been investigated as a model organism.^145^ The genome was sequenced and analysis of the FlyBase identified many of the genes expected from a SL biosynthetic pathway including SPT1 and SPT2, although a number of other genes have so far not been annotated. Taken together, these studies of alternative model higher organisms will help to build up an evolutionary picture of the SL pathway.

### 2.8 Inhibition of SPT

**Fig. 12:**
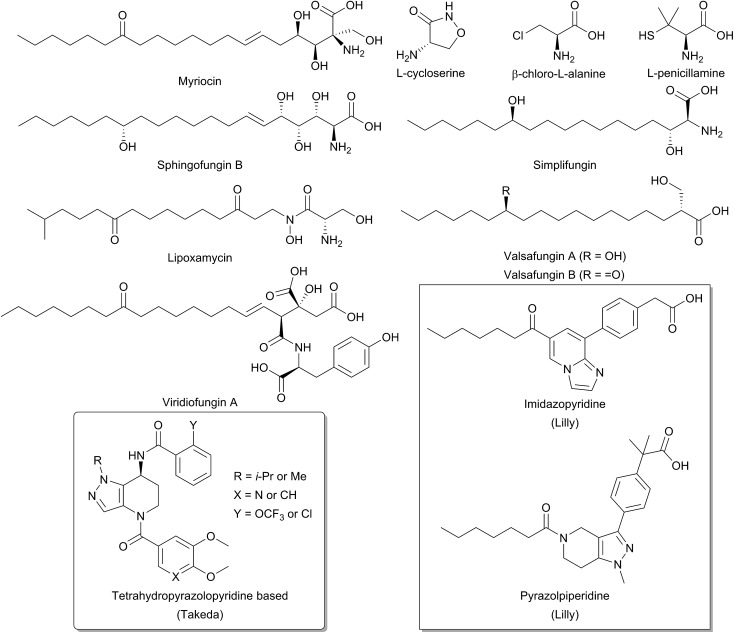
Structures of natural product and synthetic inhibitors of bacterial and mammalian SPTs. Takeda and Lilly synthetic inhibitors are highlighted in curved and square boxes respectively.

Due to the central role that it plays in the biosynthesis of SPT, and the disease states which can arise from an imbalance of SL homeostasis, SPT has been the subject of many studies to identify inhibitors. Many inhibitors of SPT directly target the PLP cofactor, and consequently are able to inhibit other PLP dependent enzymes with a broad spectrum of activity. In this review we focus on any natural products that have been shown to be potent SPT inhibitors and useful tools for SL biosynthesis in various organisms (Fig. 12).

One of the earliest SPT inhibitors identified was the mechanism-based inhibitor l-cysteine.^22^,^146^,^147^ This amino acid functions by mimicking the natural l-serine substrate; when in the active site, cysteine forms a PLP:amino acid external aldimine complex, but then readily reacts to form a ring-closed, thiazolidine–PLP compound.^21^,^148^,^149^ This results in irreversible modification of the PLP cofactor, rendering the enzyme non-catalytic. The inhibitor l-penicillamine behaves in a similar manner to cysteine by forming a thiazolidine adduct with the PLP cofactor, inhibiting the enzyme. l-Penicillamine has also been shown to inhibit other PLP enzymes such as alanine aminotransferase and glutamate decarboxylase.^150^d-Penicillamine can also bind to the PLP cofactor, but is a weaker inhibitor due to its inverse stereochemistry.^151^ Similarly, SPT can be inhibited by d-serine, which forms an external aldimine which cannot subsequently be deprotonated.^152^ Both d- and l-cycloserine are irreversible inhibitors of SPT.^45^,^153^ There is evidence that l-cycloserine inhibits SPT *via* a decarboxylative mechanism of action which forms pyridoxamine 5′-phosphate (PMP) and β-aminooxyacetaldehyde which remain bound to the active site of the enzyme.^153^

β-Chloroalanine (β-CA) is a well-known inhibitor of PLP enzymes, which readily undergoes β-elimination of the chlorine on formation of the substrate quinonoid.^154^ β-CA has been reported to inhibit a number of different PLP enzymes, including l-aspartate decarboxylase, d-alanine racemase and threonine deaminase.^154^–^157^ In Chinese hamster ovary cells, complete inhibition of SPT was seen with 5 mM β-CA in 10 minutes and the halide analogue, β-fluoroalanine can also be used to inhibit SPT.^158^,^159^ Inhibition results in the formation of a PLP-bound enamine species, which is liberated from the PLP cofactor by attack from an active site lysine. The free enamine can then react irreversibly with the lysine-PLP internal aldimine to create a covalently bound inactive PLP complex, preventing further catalysis. Halide inhibition has been reported for SPT.^53^*B. melaninogenicus* SPT was shown to be inhibited up to 60% by 25 mM NaCl. Conversely, no inhibition was seen with either sodium or ammonium acetate up to concentrations of 600 mM. Inhibition was also observed for bromide and iodide, but not for fluoride and for the pseudohalogen thiocyanate.

One feature of the natural product SPT inhibitors is their structural resemblance to sphingosine; they have a long hydrophobic tail with a polar head group. Lipoxamycin was first identified as an antifungal and antibiotic from the actinomycete *Streptomyces viginiae* in 1971 and was subsequently shown to inhibit SPT in 1994 with an IC_50_ of 21 nM against yeast SPT.^160^–^162^ Lipoxamycin demonstrated broad range antifungal activity and nanomolar toxicity against certain yeast strains such as *Candida*. Inhibition could be reversed by the addition of phytosphingosine in growth assays. Similar to lipoxamycin are a set four of related natural products from *Aspergillus fumigatus* termed sphingofungin A–D.^163^,^164^ Two further compounds, sphingofungin E and F were isolated from *Paecilomyces variotii*.^165^ Sphingofungins A, B and C were shown to be the most effective compounds, with broad spectrum activity. Sphingofungin B was the most potent, with an IC_50_ of 20 nm against *S. cerevisiae*.

Viridiofungins are a family of amino alkyl citrates (where the amino acid is either tyrosine, phenylalanine or tryptophan) isolated from *Trichoderma viride.*^166^,^167^ Viridiofungins are broad-spectrum anti-fungal agents, exhibiting nanomolar potency against *Candida albicans*, although potency against other fungi such as *S. cerevisiae* was in the micromolar range, with viridiofungin B being the most potent. Some structure–activity-relationship (SAR) analysis has been performed on viridiofungins and alterations to the acyl chain length, functional group at C13 and oxidation state at C5/C6 did little to alter the potency of the compounds.^167^ Conversely, the citric acid head group and amino acid group were both required for activity. Removal of the C1 methyl ester and the amino acid group resulted in a 30-fold and 300-fold fall in activity, respectively. However, the mechanism by which the viridiofungins inhibit SPT is unknown.

The most widely used and studied natural product SPT inhibitor is myriocin (also known as thermozymocidin). Myriocin was first isolated in 1972 from *Myrioccum albomyces* and *Mycelia sterilia* and shown to have anti-fungal activity. It was then “re-isolated” from *Isaria sinclairii* due to its potent immunosuppressive activity against lymphocyte proliferation in mouse allogeneic mixed lymphocyte reactions (MLR).^168^–^171^ Also, against mouse cytotoxic T lymphocyte cell lines (CTLL-2), myriocin was discovered to have an IC_50_ of 15 nM and was found to exclusively target SPT.^172^,^173^ SAR analysis by Fujita *et al.* showed that the C14 carbonyl group is not required for activity.^171^ Reduction of this carbonyl group to an alcohol did not affect activity, and its removal in fact increased potency. Lactonisation of the C4 hydroxyl onto the carboxyl group did not perturb activity, however, the amine group was found to be essential. Finally, hydrogenation of the C6 double bond decreased activity, as did ozonolysis of the alkene double bond. An important investigation by Schreiber and colleagues confirmed SPT to be the target of myriocin.^173^ In a pioneering study, they generated a myriocin-affinity resin by coupling the natural product *via* its acyl chain to a polymer support. Upon incubation of this “myriocin fishing hook” to CTLL-2 cells they captured myriocin-binding proteins. Mass spectrometry confirmed myriocin binds to both the SPT1 and SPT2 subunits of SPT.

Structurally related to myriocin are two new recently discovered natural product inhibitors of SPT, simplifungin and valsafungin A and B, which were isolated from *Simplicillium minatense* FKI-4981 and *Valsaceae* sp. FKH-53 respectively.^174^ All three compounds inhibited the growth of zygomycetous fungi. Derivatives of simplifungin and valsafungin were almost all less effective antifungals than the parent compounds, with the exception of a methylated derivative of simplifungin, which gave MICs as low as 0.125 μg mL^–1^ against *S. cerevisiae*. *In vitro* against yeast microsomal SPT, IC_50_ values of 224 nM and 45.4 nM were reported for simplifungin and valsafungin A. This compared with an IC_50_ of 11.8 nM for myriocin under the same conditions and of 54.4 nM for the methylated derivative of simplifungin.

**Fig. 13:**
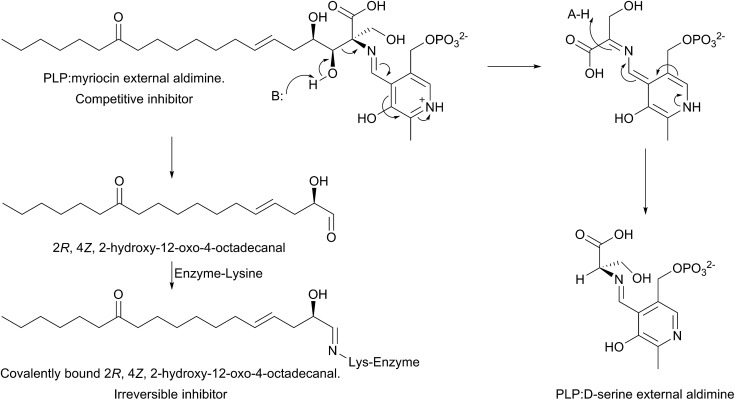
Proposed dual mechanism of inhibition of *S. paucimobilis* SPT by myriocin, in which the PLP:myriocin external aldimine undergoes a retro-aldol like cleavage, resulting in an aldehyde that covalently modifies the active site lysine (as a Schiff base) and formation of a PLP:d-serine external aldimine complex.

The mechanism of action of myriocin has been studied in some detail with SPT from various species but the most detailed mechanistic study was carried out using *S. paucimobilis* SPT^175^ (Fig. 13). At first glance it appears that the natural product functions simply by forming an SPT:myriocin–PLP external aldimine complex, in a mechanism similar to d-serine. However, although this complex does form, and the *K*_i_ (967 nM) was measured with a pure SPT for the first time, the activity cannot be restored through replacement of the PLP cofactor. Instead, it was found that SPT catalyses the slow degradation *via* an unexpected, retro-aldol like mechanism to give a PLP:d-serine external aldimine and 2*R*,4*Z*,2-hydroxy-12-oxo-4-octadecanal, which reacts irreversibly as a suicide inhibitor with the active site lysine to form an imine. This imine, derived from myriocin breakdown, was identified by mass spectrometry analysis of the inhibited SPT. Once this slow, enzyme-catalysed degradation had been discovered, it explained why X-ray diffraction quality crystals of a SPT:myriocin–PLP complex could not be obtained. Thus, a catalytically inactive SPT K265A mutant was prepared and crystals were grown in the presence of myriocin. Gratifyingly the structure of a SPT:myriocin–PLP complex was resolved but even then, the myriocin was present in a decarboxylated form. This surprising dual mode of inhibition by myriocin on the bacterial enzyme revealed that SPT is powerful enzyme that can degrade the most potent of inhibitors. Whether the same mechanism is conserved across the SPTs from various species is worthy of future study.

Recently, the results of several medicinal chemistry campaigns against SPT have been reported.^176^–^179^ Using high-throughput screening, Genin and colleagues discovered imidazopyridine and pyrazolylpiperidine based compounds as inhibitors of SPT.^176^*In vitro* imidazopyridine had an IC_50_ of 5 nM against microsomal human SPT whilst pyrazolylpiperidine gave an IC_50_ of 64 nM. In mice, application of both compounds led to a decrease in plasma ceramides, but resulted in gastric enteropathy and so could not be pursued further. Similarly, tetrahydropyrazolopyridine-derived compounds have been reported by Yaguchi *et al.* and Adachi *et al.*^177^,^178^ These compounds inhibited SPT *in vitro* and exhibit antitumor activity against acute myeloid leukaemia non-small-cell lung cancer cell lines. More detailed analysis of the mechanism of action revealed that SPT inhibition resulted in an up-regulation of COX-2 expression leading to cell death.^179^ The publication of these results suggest that SPT is a potent and viable target for novel therapeutics.

### 2.9 Regulation of SPT

The cellular flux and balance of the SL pathway is under very tight control and allows cells to respond to the supply and demand for SLs and ceramides during growth, division and apoptosis – the so-called “sphingolipid rheostat”. The links between SLs and diseases continue to grow and SL regulation has been the subject of intensive study. This area has been reviewed recently.^7^,^16^,^180^–^184^ At a molecular level, the control of the activities of the enzymes involved in the pathway (Fig. 1) play an important role, but the exact details are still unclear. Since SPT is the first enzyme, pulling substrates from the l-serine and acyl-CoA pools to generate 3-KDS, it is referred to as the committed step in the pathway and it would make mechanistic sense to have regulatory control at this key junction. The issue of SPT regulation is a complex and active area of research.

Studies in yeast and mammalian cells identified the SPT-activating Tsc3p and ssSPTs/b subunits respectively (Section 2.4) but subsequent studies have also identified additional partners that play a role in SL regulation. These proteins, the yeast ORMs (orosomucoid, ∼25 kDa) and their homologs in higher eukaryotes, the ORMDLs (orosomucoid-like) were discovered only recently. ORM proteins were first identified in yeast as transmembrane proteins localised to the endoplasmic reticulum (ER). There are three ∼17 kDA ORMDLs in mammals (ORMDL1, 2 & 3) whereas yeast have only two ORMs (ORM1 and ORM2).^185^

#### 2.9.1 ORMs in yeast

For some time after their initial discovery, the function of ORMs remained obscure and it was not until 2010 that it was shown that yeast ORMs associate with SPT, forming what is known as the “SPOTS” complex, consisting of SPT1, SPT2, Tsc3p, ORM1, ORM2 and Sac1.^10^,^186^ By analysing gene ontology data from yeast, Breslow *et al.* observed that increased levels of ORM1 and ORM2 correlated with decreased activity of SPT, implying that the ORMs are negative regulators of SPT.^187^–^189^ Further evidence for a role of the ORMs in regulating SPT came from the demonstration that they are physically associated. This was achieved by FLAG-tagging LCB1 and performing pull-down experiments.^10^ The isolated proteins were identified by mass spectrometry. In contrast to the ORMs, the role of the SPOTS partner, Sac1 – a phosphoinositide phosphatase, is somewhat mysterious. Sac family proteins have roles in numerous cell functions, such as membrane trafficking, however, the role of Sac1 in SL homeostasis and the reasons for its association with SPT and the ORMs is unknown.^190^ Using a different approach, Han *et al.* used ORM1 and ORM2 yeast knockouts and observed a loss of SL homeostasis following deletion of these genes. Since this phenotype could be reversed by myriocin inhibition of SPT, it suggested that the ORMs negatively regulate the activity of SPT. Additionally, overproduction of ORM2 in yeast results in decreased levels of SL biosynthesis.^191^

**Fig. 14:**
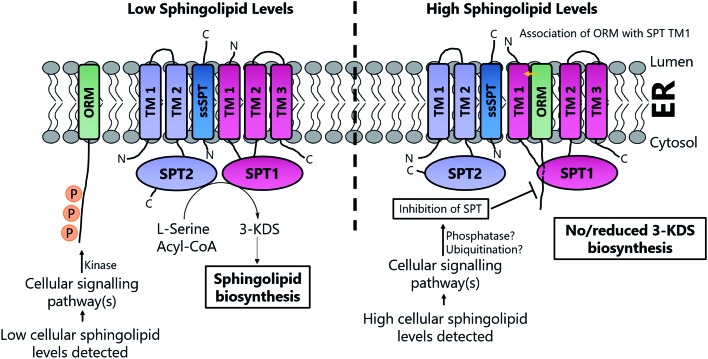
Proposed mechanisms of regulation of SPT under high and low cellular SL levels in yeast. At low cellular SL concentrations, the ORM proteins are phosphorylated by a kinase, which prevents association with and inhibition of SPT. However, under high SL conditions, the ORMs are non-phosphorylated, allowing interaction with TM1 of SPT1, inhibiting 3-KDS biosynthesis.

Based on the work of Han *et al.* and Breslow *et al.*, a putative, basic working model of SPT regulation has emerged^10^,^186^ (Fig. 14). At high SL levels, SPT1, SPT2, Tsc3p, Sac1, ORM1 and ORM2 exist in the “SPOTS complex” where association with the ORMs results in inhibition of SPT activity, thereby blocking *de novo* SL biosynthesis. The precise molecular mechanism by which the ORMs inhibit SPT activity is unknown. However, the ORM proteins have no known catalytic function, so inhibition may result directly from binding. In yeast, the affinity of the ORMs for SPT is regulated by phosphorylation of their N-termini. At low SL levels, phosphorylation of the ORM proteins results in their dissociation from the SPOTS complex, releasing SPT and allowing SL biosynthesis to occur. Phosphorylation occurs at the N-terminus of the ORM proteins and this leads to derepression of SPT, most likely by causing a conformational change in the ORM protein. Since Sac1 is a known phospho-lipid phosphatase which has been implicated in the control of actin cytoskeleton and vacuole morphology, it is unclear what direct role, if any, it has in SL homeostasis.^192^,^193^

The mechanisms by which the ORM proteins themselves are regulated is also an active and complex area of research. However, it is known that the ORMs are phosphorylated through a kinase cascade involving Ypk1, TORC2 194 and TORC1,^195^,^196^ possibly integrating signals from a number of different pathways to control SL levels. Additionally, bioinformatic studies have shown correlations between the ORMs and other proteins, an example being the morphogenesis checkpoint kinase Swe1.^197^,^198^ In yeast, Swe1 is able to regulate SL biosynthesis independently of Ypk1, presumably *via* phosphorylation of the ORM proteins.

#### 2.9.2 ORMs in higher eukaryotes

ORMDL family proteins regulate SL biosynthesis in mammals and associate with SPT in a similar manner to ORMs in yeast.^10^ ORMDLs came to prominence when an association between asthma and single nucleotide polymorphisms in the region adjacent to the human ORMDL3 gene locus were reported in 2007.^183^ These SNPs, associated with an increase in the expression of ORMDL3 mRNA, are proposed to increase the risk to asthma through perturbation of SL homeostasis.^183^,^199^–^201^

Like their cousins from yeast, the mammalian ORMDLs are small, hydrophobic, transmembrane proteins approximately 17 kDa in size.^185^ However, unlike the ORMs, the ORMDLs do not have an N-terminal tail region whose phosphorylation regulates their inhibitory capacity. Consequently, the mechanisms by which the ORMDLs control SL biosynthesis differ from the ORMs. ORMDLs are functionally redundant and deletion of all three isoforms is required to entirely abolish SPT regulation.^202^,^203^ Similarly, it has been reported that overexpression of all three ORMDL isoforms in HEK293 cells is required to inhibit SPT activity.^204^

In a series of elegant experiments to investigate the regulation of ORMDLs and SPT, Gupta *et al.* have made use of an SPT overexpression cell line.^205^ Upon induction of SPT activity in an HEK cell line, increased ORMDL expression is observed. ORMDL induction is dependent upon SPT activity since overexpression of a catalytically inactive SPT (SPT2 lysine mutant) failed to induce expression of the ORMDLs, as did addition of myriocin. As such, a product of SPT is in part responsible for ORDML regulation. Gupta *et al.* also noted that inhibition of ceramide synthase activity *via* the inhibitor fumonisin also reduced levels of ORMDL. This supports observations by Siow and Wattenberg that a complex SL downstream of ceramide is responsible for ORMDL induction.^202^

Based on published data, it is apparent that ORMDL expression is not regulated by one single factor but through a complex regulatory mechanism which is still being deciphered, although there is as yet no evidence for any post-translational modifications.^205^ Beyond the observation that ORMDL expression changes in response to an as-yet unknown complex SL, it is unclear whether each ORMDL isoform is responding to the same signal or whether ORMDL control of SPT simply occurs by binding to the enzyme.

An additional layer of complexity is added by phosphorylation sites on human SPT. Taouji *et al.* identified Tyr164 as a phosphorylation site of SPT1.^206^ Phosphorylation of Tyr164 is mediated by the tyrosine kinase ABL and increases SPT activity. Another such phosphorylation site has been identified on the SPT2 subunit, Ser384.^113^,^125^ Phosphorylation of Ser384 caused a decrease in activity towards l-serine. However, increased activity towards l-alanine was also observed. The physiological significance of SPT phosphorylation remains to be determined.

## 3 3-Ketodihydrosphingosine reductase (KDSR)

**Fig. 15:**
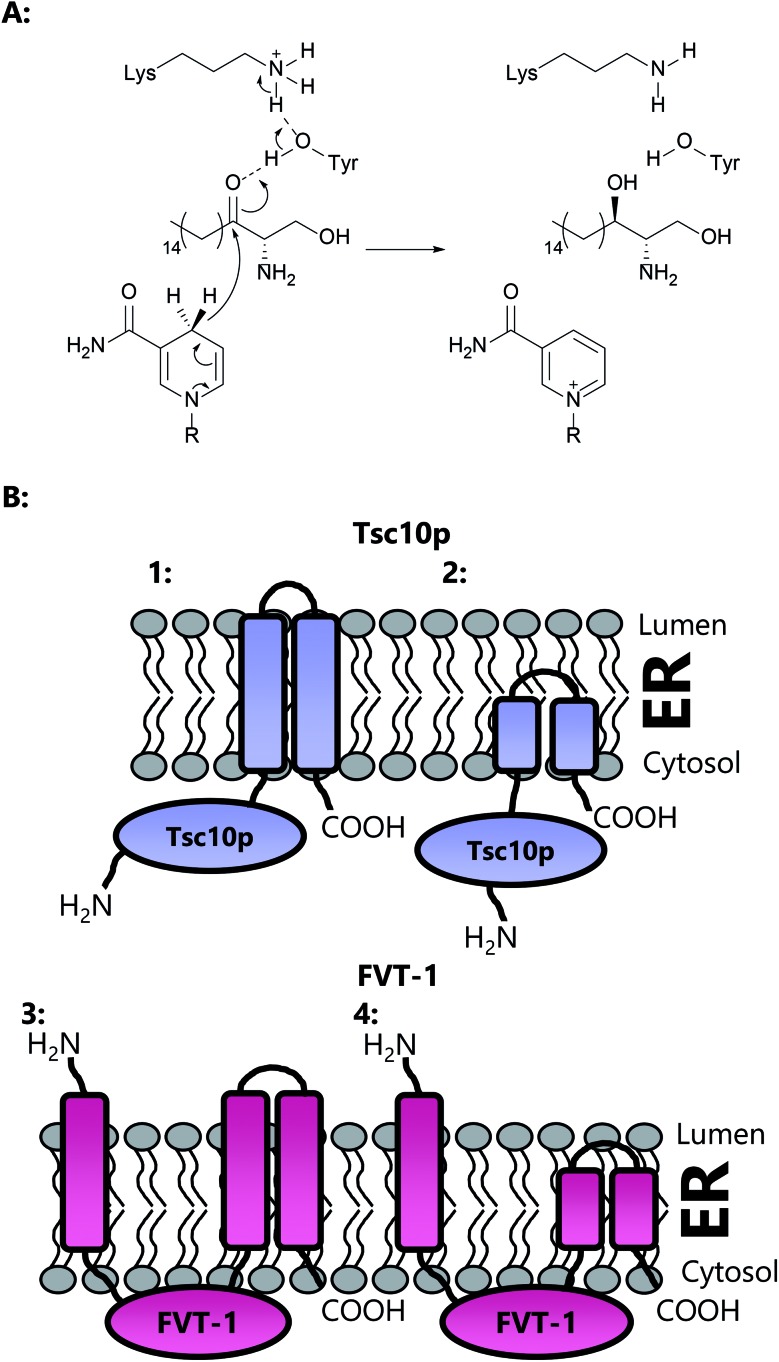
(A) Proposed mechanism of the NADPH-dependent, KDSR-catalysed reduction of 3-KDS that converts the 3-keto group of 3-KDS to give DHS. (B) Proposed topologies of Tsc10p (1 & 2) and FVT-1 (3 & 4) within the ER membrane.

The second enzyme on the committed pathway to SL biosynthesis is 3-ketodihydrosphingosine reductase (3-KDSR), which catalyses the NADPH-dependent reduction of 3-KDS to give sphinganine (2*S*,3*R*-dihydrosphingosine (DHS)) (Fig. 15a). 3-KDSR was first identified by Dunn and co-workers in *Saccharomyces cerevisiae* using a temperature/calcium sensitivity screen looking for genes encoding enzymes involved in SL biosynthesis.^207^ Tsc10p was found to belong to a large superfamily of enzymes known as the short chain dehydrogenases/reductases (SDR) and when expressed *in vitro* was shown to catalyse the NADPH dependent reduction of 3-KDS. Tsc10p has been subsequently characterised in two other microbial strains, *Candida albicans* and *Aspergillus fumigatus*, and identified in a number of different strains through sequence similarity.^208^ KDSR has been identified in *Arabidopsis*,^209^ and using yeast complementation assays, a Tsc10p homolog has been found in mammals, FVT-1, which exhibits 24% identity and 42% similarity with Tsc10p.^210^

Yeast Tsc10p (320 aa, ∼36 kDa) contains a 28 amino acid C-terminal stretch which is proposed to anchor and direct the enzyme to the ER membrane (Fig. 15b). Removal of this C-terminal stretch is not deleterious to protein activity, suggesting that Tsc10p contains a hydrophilic N-terminal catalytic domain. Human FVT-1, on the other hand, is proposed to contain three trans-membrane regions, with the N-terminal trans-membrane region facing the ER lumen and the C-terminus facing the cytosol. The transmembrane N-terminal region is required for targeting of FVT-1 to the ER membrane. A large hydrophilic domain, which contains the proposed active site, is thought to face the cytosol. It has been suggested, however, that the transmembrane regions in FVT-1 and Tsc10p may not span the entirety of the membrane, and may merely constitute a hydrophobic domain which embeds into the membrane.^211^ Both Tsc10p and FVT-1 are multimeric, as shown by immunoprecipitation experiments.

All four functionally characterised KDSRs (*S. cerevisiae*, *H. sapiens*, *C. albicans* and *A. fumigatus*) share conserved serine, threonine and lysine residues (based on *S. cerevisiae* numbering; Ser167, Tyr180 and Lys184), corresponding to the catalytic triad of residues found in SDRs and the TGxxxGxG motif at the N-terminus for coenzyme binding.^212^,^213^ Oppermann and colleagues have shown that SDRs likely have, in fact, a catalytic tetrad incorporating Ser167, Tyr180, Lys184 and Asn140.^214^ However, sequence alignment of the four enzymes discussed here shows that the Asn140 residue is not conserved in *A. fumigatus* (replaced with a valine). Using the structures of related short chain dehydrogenases, Fornarotto and co-workers have constructed a homology model of 3-KDSR from *A. fumigatus*.^208^ Docking of the 3-KDS and NADPH substrates into the homology model suggests that the majority of the 3-KDS substrate is not involved in binding to the enzyme active site, and protrudes into the solvent. The enzyme active site is proposed to be at the C-terminus of the enzyme, close to the membrane associating helix, allowing the hydrophobic substrate to embed within the membrane. Given this potentially broad substrate specificity, a variety of shortened 3-ketosphinganine analogues were screened against *A. fumigatus* 3-KDSR. Interestingly, C_8_, C_11_ and C_12_ analogues were all turned over by the enzyme (monitoring NADPH consumption), albeit with diminished *K*_m_ and *k*_cat_ values.

Yeast Tsc10p knockouts are not viable, indicating that Tsc10p is the only 3-KDSR in the yeast genome. However, in mammalian genomes, the presence of orphan SDRs means that FVT-1 may not be the only 3-KDSR enzyme present. Interestingly, siRNA experiments in CHO cells indicate that FVT-1 is the major enzyme responsible for 3-KDSR activity in mammals. Given the differences in topology between FVT-1 and Tsc10p, it is remarkable that FVT-1 is able to rescue Tsc10p knockouts, and suggests there are several mechanisms in yeast for targeting proteins to the ER membrane.^211^ A detailed mechanistic understanding of how any KDSR controls the stereochemical outcome that leads to formation of the 2*S*,3*R* diastereomer of DHS will require a high resolution crystal structure of the enzyme:substrate complex. Moreover, since the discovery of the deoxy-SLs, it is apparent that the KDSRs and subsequent downstream enzymes can accept deoxy derivatives of 3-KDS for reduction. Of note is the apparent lack of association between SPT and KDSR. SPT immunoprecipitation experiments have not pulled down KDSR,^10^ so it is unknown whether SPT simply deposits 3-KDS into the membrane for KDSR ‘to find’, or whether there is some channelling of the substrate.

### 3.1 Disease associated mutations in KDSR

Mutations in the KDSR gene have been associated with a small number of disease states in cattle and humans. In cattle, bovine spinal muscular atrophy (SMA) is a recessive neurodegenerative disease which causes loss of motor neurons.^215^ Mapping of the SMA locus led to the FVT-1 gene, where a missense G-to-A mutation was found, resulting in a A175T mutation. *In vitro* analysis of the recombinant bovine FVT-1 protein (truncated by the first 25 residues to remove a putative transmembrane domain) revealed that the mutant enzyme displays no activity. Interestingly, in an *S. cerevisiae* tsc10Δ knockout, expression of FVT-1 and the FVT-1 A175T mutant resulted in the same growth phenotype, indicating that although no activity can be detected *in vitro*, *in vivo* there may be some residual activity. Although the molecular cause of this loss of activity is unknown, bovine Ala175 is very close to Ser173, which correlates to Tsc10p Ser167, a conserved residue of the catalytic triad. As such, the bovine A175T mutation may disrupt the catalytic triad, preventing catalysis.

In humans, mutations in KDSR have recently been associated with an array of keratinization disorders (skin disorders).^216^,^217^ These two separate studies performed exome sequencing and found a variety of different mutations in the KDSR gene, including gene inversions, base pair deletions and substitutions. The deletion mutations identified likely lead to exon skipping or frame shifts, resulting in catastrophic effects on the protein structure. In one case, the conserved catalytic tyrosine is mutated (Y186F), preventing catalysis. *In vivo* assays using an *S. cerevisiae* tsc10Δ knockout showed that expression of the majority of the mutant genes failed to complement the tsc10 knockout, demonstrating the deleterious effects of these mutations on enzyme activity. The only exception was a F138C mutation, which was found to have a mild effect *in vivo.* Takeichi *et al.* analysed a number of the mutations *in vitro* using microsomal preparations of HEK293 cells. In a similar result to the *in vivo* yeast assay, all but one mutation abolished KDSR activity. Interestingly one mutation, G182S, did not lead to a significant loss in DHS production, which is unexpected given that Gly182 lies in a hydrophobic domain in close proximity to the active site tyrosine and would be predicted to lead to structural changes in the protein. However, without a high resolution structure of human KDSR, it is difficult to explain conclusively in molecular terms the effect of these mutations on the catalytic activity.

## 4 Sphingosine kinase (SK)

Sphingosine kinases (SKs) are ATP dependent kinases of the diacylglycerol kinase (DAGK) family, which phosphorylate the terminal hydroxyl group of sphingoid bases – either sphinganine (from *de novo* synthesis) or sphingosine (from ceramide breakdown). Sphingosine, the long chain base derived from ceramide hydrolysis, is phosphorylated by SK to give sphingosine 1-phosphate (S1P). S1P is an important signalling molecule both inside and outside of the cell, where it binds to S1P-specific G-protein coupled receptors triggering a cascade of SL-dependent cellular events.^16^ Given its central role as a second messenger, changes in S1P homeostasis have been linked to diseases such as cancer and diabetes.^218^–^222^ The biological function and regulation (for example palmitoylation and phosphorylation) of the protein of S1P and SK is a large, complicated and intensively active area of current research.^223^ As such, these topics will not be discussed here. Rather the interested reader is directed to several excellent reviews in the area.^224^–^227^

In mammals, SK is encoded by two genes, each of which give rise to multiple splice variants,^228^–^230^ which differ in the length of their N-terminal domain.^229^,^231^–^233^ Whilst SK1 (also termed SPHK1) is mostly cytosolic, SK2 (also termed SPHK2) is predominantly found within the nucleus and has a large insert in the lipid binding domain.^234^–^237^ The SK isoforms also differ in their tissue distribution and in their substrate specificities. Although both have similar specificities for the natural d-*erythro*-sphingosine substrate (*K*_M_ of 3.4 μM), SK2 has an increased specificity for d-*erythro*-dihydrosphingosine, dl-*threo*-dihydrospingosine and phytosphingosine. Conversely, d-*erythro*-sphingosine was the best substrate for SK1.^230^ Neither SK1 nor SK2 phosphorylates ceramides but a specific ceramide kinase has been discovered^238^ and cloned.^239^,^240^ SK1 has also been reported to accept GTP as a nucleotide substrate as well as ATP, albeit with a significantly reduced preference^241^ The immunosuppressive drug FTY720 (Fingolimod, Gilenya™, see below) sold by Novartis to treat multiple sclerosis is, in fact a “pro-drug” that is phosphorylated by SK1 and SK2.^233^,^242^,^243^

Despite this difference in substrate preferences, SK1 and SK2 are functionally redundant.^230^,^244^ Either SK1 or SK2 can be knocked out in mice, and result in viable progeny.^245^,^246^ A double SK1 and SK2 mutant however, is not viable.^246^ Interestingly, overexpression of either SK1 or SK2 in mice has opposing effects. Overexpression of SK2 increased incorporation of [^3^H]-palmitate into C_16_ ceramide, whereas overexpression of SK1 decreased incorporation.^236^ This indicates that the two SK isoforms have contrasting functions in the homeostasis of ceramide biosynthesis. SK homologs have been identified in *Leishmania major* and *Trypanosoma brucei*.^247^,^248^ In *T. brucei* SK has been shown to be important in controlling the cell cycle whilst in *L. major*, SK is responsible for detoxification of sphingoid bases and regulating the stress response.

### 4.1 Structure of SK1 (SPHK1)

**Fig. 16:**
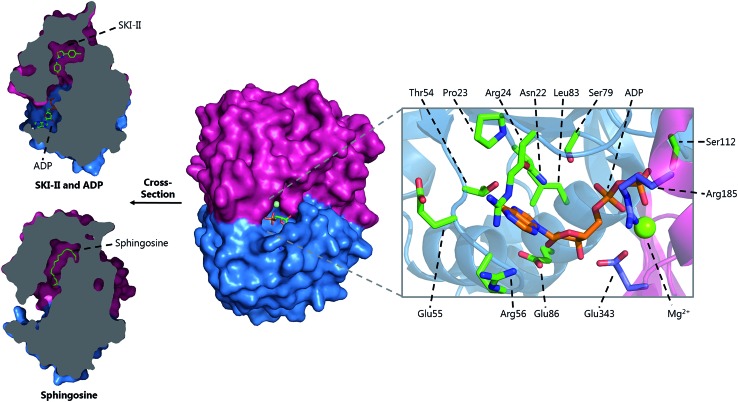
3D structure of human SK1 homodimer with the inhibitor SKI-II/ADP bound (PDB: 3VZD) and sphingosine bound (PDB: ; 3VZB). The ADP binding site is highlighted, showing the residues required for substrate binding, provided by both subunits.

The SK proteins are unusual amongst the SL biosynthesis enzymes as they are not integral membrane proteins. Rather, SKs associate with the plasma membrane when activated *via* a process which is related to their regulation (reviewed in Pyne *et al.*^224^). Despite being cloned in 2000 228 it was surprising that the crystal structure of human SK1 was only reported by Amgen in 2013.^249^ The overall 3D structure was novel and bears no similarity to other previously-characterised protein or lipid kinases. The structure revealed a two domain enzyme split into N- and C-terminal halves (Fig. 16).^249^ The C-terminal domain consists of the sphingoid base binding site and has a mainly antiparallel β-sandwich architecture. The sphingosine substrate is proposed to bind in a J-shaped configuration, with the polar head group held in place in the inter-domain cleft through hydrogen bonding with Asp81 and Ser168. Wang *et al.* reported electron density which could not be assigned to detergents used in crystallography, and thus it is suggested that this density is due to a C_16_ long-chain base retained by the enzyme through purification. The hydrophobic tail of sphingosine then sits in a J-shaped hydrophobic pocket. This hydrophobic pocket is, however, almost completely buried within the enzyme, and is only accessible through a hydrophilic opening in which the sphingosine head group sits. Comparison of the structures of the substrate-bound and ligand-free SK1 structures reveals a side opening in the enzyme. Wang *et al.* also suggest that two α-helices (α7 and α8) might act as a gate, and control binding of the sphingosine substrate through this side gate, allowing access to the active site.

The catalytic site of SK1 is found at the cleft between the N- and C-terminal domains. The N-terminal domain itself consists of an α/β fold and encompasses the binding site for the nucleotide substrate. Crystals grown in the presence of ADP reveal that the nucleotide binds in this cleft and the β-phosphate of ADP bridges the N- and C-terminal domains. A multitude of interactions are required for binding, including hydrogen bonding, π-stacking interactions and coordination to a Mg^2+^ cofactor.^249^,^250^ A recent study by Pulkoski-Gross *et al.* used hydrogen–deuterium exchange mass spectrometry to identify a positively charged motif on the surface of SK1 which is responsible for binding to membranes *via* anionic phospholipids, and regulating the correct function of the enzyme.^251^

### 4.2 Mechanism and inhibition of SKs

**Fig. 17:**
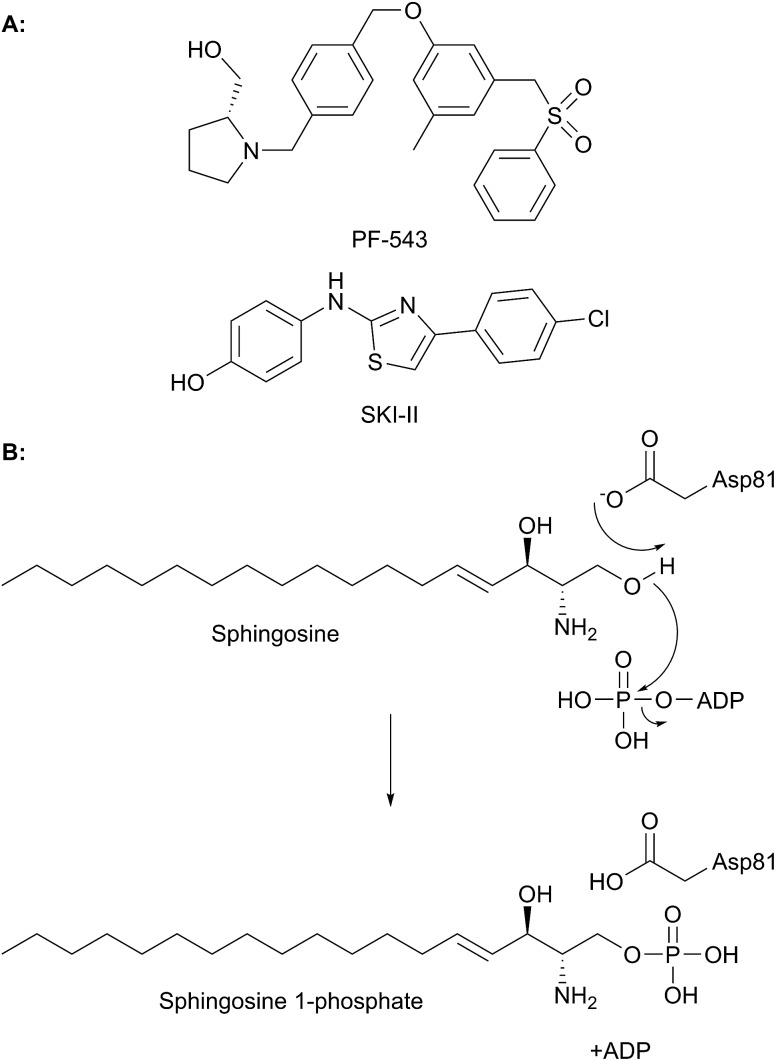
(A) Structures of the SK inhibitors PF-543 and SKI-II. (B) Mechanism of phosphorylation of sphingosine by SK1.

Since SK1 is a drug-discovery target, the determination of the crystal structure of human SK1 helps rationalise the medicinal chemistry programmes that seek to exploit this key enzyme. A number of small molecule compounds have been reported as inhibitors of SKs (as reviewed by Santos & Lynch,^252^ Sanllehí *et al.*^253^ and Pitman *et al.*^254^), the majority of which target the sphingosine binding site. Due to the large number of inhibitors reported, we will only focus on those whose structures have been characterised in complex with SK1. A number of sphingosine-like compounds act as inhibitors including *N*,*N*-dimethyl-sphingosine (DMS)^241^,^255^ and dl-*threo*-dihydrosphingosine.^256^,^257^ However, other small molecules, not structurally related to sphingosine have also been reported.^225^,^252^ SK1 was first crystallised in the presence of the inhibitor SKI-II (Fig. 16a), a known SK1 and SK2 inhibitor with antitumor activity.^249^,^258^,^259^ SKI-II binds in the hydrophobic pocket of SK1, where the hydrophobic chain of a lipid (believed to be sphingosine) is bound, suggesting SKI-II is a competitive inhibitor of the SKs. The inhibitor is held in place by a series of hydrogen bonds, van der Waals interactions and hydrophobic interactions. A crystal structure in the presence of PF-543 (Fig. 17a, the most potent SK inhibitor reported to date) has been reported.^237^,^260^ Like SKI-II, PF-543 binds in the lipid binding pocket of the enzyme in a bent conformation. This bent orientation somewhat resembles that of the “sphingosine” reported in the structure of SK1 by Wang *et al.*^249^ Likewise, PF-543 binds through a series of hydrophobic interactions and hydrogen bonds. Interestingly, the hydroxymethyl moiety of PF-345 binds adjacent to the ATP binding site, apparently mimicking the polar head group of sphingosine.

Mechanistically, SK catalyses the transfer of the γ-phosphate from a molecule of ATP onto the C1 hydroxyl group of sphingosine (Fig. 17b). The catalytic mechanism begins with deprotonation of the C1 hydroxyl, which then attacks the phosphate group of ATP, giving ADP and phosphorylated sphingosine as products. Some questions remain with regards to the base responsible for deprotonation with Asp81 proposed to play this role.^249^ In addition, Asp81 is also believed to be involved in hydrogen bonding to the cap of helix α5. Involvement of Asp81 may require movement of the enzyme during substrate binding and catalysis.^225^ With the 3D structure of the human SK1 in hand, we would expect similar molecular analysis of SL kinases from other species to follow. As well as laying a foundation for future drug discovery, the SK structures will also provide insight into kinase regulation and translocation by factors such as calmodulin.^261^

## 5 Sphingosine 1-phosphate lyase (S1PL)

Both SPT, the first enzyme that controls entry to SL biosynthesis, and sphingosine-1-phosphate lyase (S1PL), the final enzyme that regulates exit from the SL pool, are PLP-dependent enzymes. S1PL catalyses the retro-aldol like cleavage of S1P to hexadecenal (2*E*-HEX) and phosphoethanolamine (PEA), both of which are substrates which feed into glycerophospholipid synthesis^262^ and the former of which has been demonstrated to have signalling function (Fig. 2).^12^,^263^ In theory, this S1PL-dependent reaction is reversible, but to date, no conversion of 2*E*-HEX and PEA to S1P has been described. This effectively makes the S1PL-catalysed metabolism of S1P irreversible, acting as a release valve to maintain a SL balance.

S1PL, like SPT, is a type 1 PLP enzyme first identified in *S. cerevisiae* (where it is termed Dpl1p and BST1) by Saba *et al.* in 1997, with identification of the human homologue following three years later.^12^,^220^ In yeast, S1PL function has been implicated in a variety of different processes such as heat stress and nutrient deprivation.^264^,^265^ Deletion of S1PL in yeast by Saba was found to result in a severe sensitivity to sphingosine, with cellular growth inhibited at micro molar concentrations of exogenously added sphingosine, likely due to the inability of the deletion strain to remove SL from the cell.^220^

**Fig. 18:**
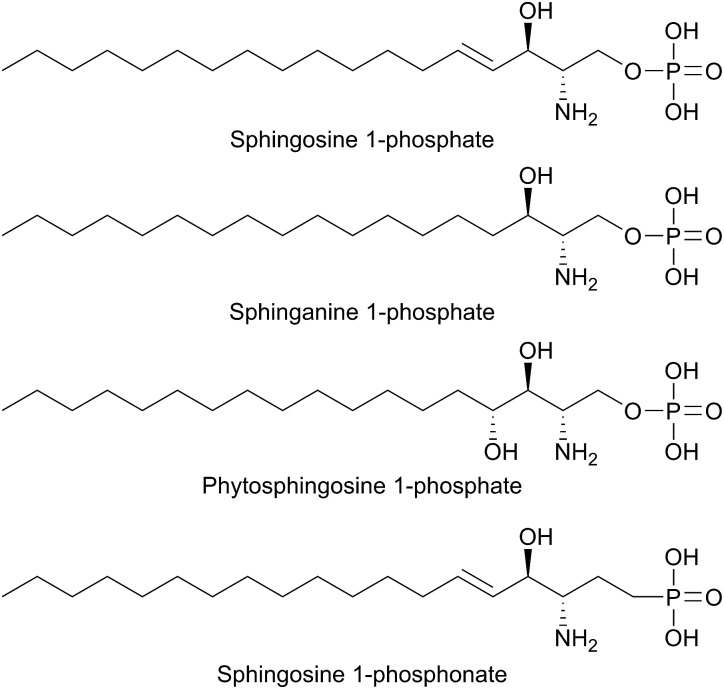
Structures of the S1PL substrates sphingosine 1-phosphate, sphinganine 1-phosphate, phytosphingosine 1-phosphate and sphingosine 1-phosphonate.

Human S1PL shows a high degree of substrate stereospecificity (Fig. 18), only accepting substrates of d-(+)-*erythro* conformation.^12^ This stereospecificity likely reflects the need to correctly position the C2 amine to react with PLP and to position the C3 hydroxyl to interact with an active site base. However, S1PL from rat liver extracts is significantly less regiospecific and can cleave a variety of different sphingoid bases including S1P, sphinganine 1-phosphate, phytosphingosine 1-phosphate and sphingosine 1-phosphonate.^266^ However, S1PL cannot turnover sphinganine. As such, the phosphate group is essential for binding. However, modifications to the head group are tolerated. For example, sphinganine 1-phosphonate is also accepted by S1PL in rat liver extracts, but at a diminished rate,^267^ indicating that both the phosphate head group and the hydrophobic tail are necessary, but not sufficient, for binding. Mouse S1PL is tolerant towards modifications to the acyl chain, such as C4 or C5 methylation.^268^ In the case of S1P, the geometry of the C4 olefin did not alter activity. Activity towards substrates with acyl chain lengths as short as C_7_ and as long as C_20_ has been reported.^269^,^270^

Given the important functions that S1P performs in the cell, S1PL therefore has an important role in controlling cellular levels of this signalling molecule. However, understanding the biochemistry of yeast and mammalian S1PL homologs is hampered by their association with the ER membrane and the insolubility of substrate. A soluble homologue of S1PL is present in the thermophilic bacteria *Symbiobacterium thermophilum*.^271^ Sequence alignment of the *S. thermophilum* with other S1PL sequences (yeast, human) reveals that it lacks the transmembrane domain, likely contributing to its solubility.

### 5.1 Structure of S1PLs

**Fig. 19:**
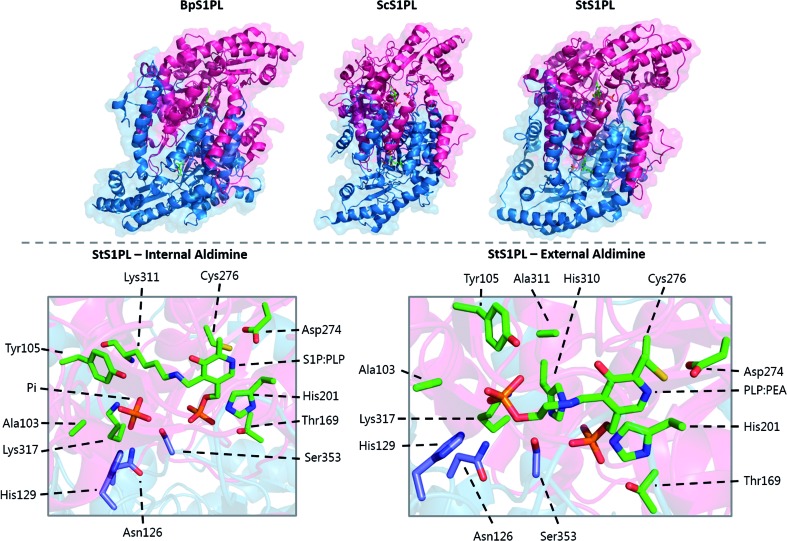
3D structures of S1PLs from *B. pseudomallei*, *S. cerevisiae* and *S. thermophilum.* The internal aldimine (PLP cofactor covalently bound to Lys311) and product external aldimine (PLP–PEA non-covalently bound to the K311A mutant) forms of *S. thermophilum* are also shown, highlighting the residues involved in substrate binding and catalysis.

Taking advantage of its inherent solubility, the *S. thermophilum* S1PL homolog (StS1PL) was the first to be crystallised (Fig. 19). It is a homodimeric enzyme, displaying a characteristic 3D fold shared with other type I PLP enzymes.^271^ The structure of an StS1PL subunit can be divided into four regions: an N-terminal domain (which is disordered, termed the Nt-FLEX domain), a central catalytic domain, a C-terminal domain and a C-terminal extension domain. As is the case with SPT, the active site is found at the subunit interface and residues from both subunits are required to form the active site. Interestingly, two conformations of StS1PL were crystallised. One structure was symmetrical whilst the other asymmetrical. In the asymmetrical St1SPL conformation, one of the active sites lacks the PLP cofactor, whilst in the symmetrical StS1PL, both active sites contain the PLP cofactor. Moreover, the asymmetric and symmetric conformations had varying levels of disorder within the C-terminal extension, with the C-terminal domain in the asymmetric StS1PL being completely disordered. This asymmetric form is somewhat similar to human and yeast SPT, in which there is believed to be only one active site (see Section 2.0).

The enzyme active site is centred around the PLP cofactor and contains residues from both subunits. A long hydrophobic tunnel extends from the surface of the protein to the active site. Specifically the PLP cofactor forms an imine with lysine 311 and the pyridine ring is positioned between histidine 201 and cysteine 276 (Table 3, Fig. 19). The PLP phosphate is bound by a classical phosphate binding cup, again comprising residues from both subunits. A crystal structure of the K311A StS1PL mutant with the product PEA reveals that a pocket of residues comprising Tyr105 and Ala103 of one subunit and Asn126 and His198 from the adjacent subunit is required for binding of the phosphate of the substrate.

**Table 3:** Summary of key residues in S1PL enzymes across different species. Residues marked with * are provided by the adjacent subunit

(Proposed) role	Organism				
	StS1PL	Dpl1p	HsS1PL	PlS1PL	BsS1PL
PLP phosphate binding cup	G168	G235	G210	G220	G126
	T169	T236	T211	T221	T127
	H310	H379	H352	H359	H270
	S353*	S422*	S395*	S402*	S313*
PLP sandwich	C276	C344	C317	C328	C235
	H201	H268	H242	H252	H159
Substrate phosphate binding	Y105	Y174	Y150	Y160	Y58
	H129*	H198*	H174*	H185*	Q82*
	A103	A172	T148	A157	T56
	N126*	N195*	N171*	N182*	N79*
	K317	K386	K359	K366	K277
PLP binding	K311	K380	K353	K360	K271
PLP aspartate	D274	D342	D315	D326	D233
Substrate entry	Y482	Y554	Y526	Y543	Y442

In *S. cerevisiae*, Dpl1p is bound to the ER membrane by a single-pass transmembrane domain on the N-terminus of the protein.^266^,^272^,^273^ The N-terminal domain faces the ER lumen and is glycosylated, whilst the active site is located on the cytosolic face of the protein in a large soluble domain. Dpl1p forms hetero-oligomeric structures. Interestingly, the formation of these SPL oligomers is dependent upon the transmembrane region. Removal of the transmembrane region similarly results in a loss of protein activity. This is likely due to the fact that the active site is at the dimer interface, utilising residues from both subunits.^260^

The structure of Dpl1p (lacking the first 102 residues corresponding to the transmembrane region, termed Dpl1p Δ1–102) was reported at the same time as the structure of StS1PL.^271^ Analysis of the Dpl1p structure reveals that it is strikingly similar to the *S. thermophilum* enzyme, and shares the disordered N-terminal domain. The analogous residues to those found in StS1PL involved in the PLP phosphate binding cup, PLP binding and substrate binding are listed in Table 3. Interestingly, Dpl1p Δ1–102 was inactive *in vitro*, which is believed to be due to the disorder on the C-terminal extension. It is proposed that the transmembrane domain (residues 1–102) is required for stability of the C-terminal extension, which is in turn required for substrate penetration to the active site.

Mutation of certain key yeast S1PL residues identified in the crystal structure (K380, Y174, A172, H198, C344, K386 and Y554) believed to be required for function has no effect on the viability of yeast in *in vivo* phytosphingosine sensitivity assays.^271^ However, mutation of residues Lys380, Ala172, and Lys386 was found to abolish S1PL activity, preventing yeast growth. Residue Lys380 is required for PLP binding, and so it is unsurprising that it is essential for activity. Ala172 is proposed to be involved in binding of the substrate phosphate moiety. Bourquin *et al.* mutated this residue to a proline, resulting in inactivated protein. It is unclear whether this mutation is in fact essential for activity, or whether the proline residue could possibly have introduced a turn into the structure, thus resulting in a misfolded, inactive protein. Lys359 meanwhile is believed to electrostatically contribute to binding of the substrate phosphate moiety. Consequentially mutation of this residue may prevent substrate binding. Mutation of residues Cys344, Tyr174, His198 and Tyr554 was found to be less deleterious, but resulted in significantly less healthy yeast. Tyr554 is believed to be important for substrate accommodation in a hydrophobic pocket, whilst His198 and Tyr174 are required for substrate binding. Cys344 is believed to perform a structural role, assisting in maintaining the orientation of the PLP cofactor.

Homologs of S1PL have been discovered in various other organisms including the microbial species and intra-cellular pathogen *Legionella* and the trypanosomatid protozoan *Leishmania*. In *L. major*, deletion of S1PL allows the organism to grow, but it is defective in stationary phase and virulence.^274^ In contrast to *Leishmania*, which produce sphingolipids and have the enzymes required for SL biosynthesis,^275^*Legionella* appears to lack SLs and the other genes of the *de novo* SL biosynthetic pathway. Therefore, this observation raises the question as to what role(s) SLs play in these organisms. The *L. pneumophila* S1PL (LpS1PL) displays sequence homology with the yeast (28.4 identity, 43.7% similarity), human (30.2% identity, 46% similarity) and *S. thermophilum* (27.9% identity, 41.2% similarity) enzymes and was isolated by Rolando *et al.*^276^ In LpS1PL, mutation of residues Cys328 and lysine 366 resulted in decreased enzyme activity. Cys328 is analogous to Cys344 in Dpl1p whilst Lys366 is analogous to Lys380. It is interesting that the lysine residue is essential for activity in Dpl1p whilst it is not essential in LpS1PL. A structure of the enzyme, albeit without the essential PLP cofactor, allowed comparison of the bacterial and human S1PLs. This study also showed that *Legionella* can reduce the host SLs in a S1PL-dependent mechanism. This work suggests that in pathogens S1PL plays a role in virulence and provides a link between SLs and autophagy.

The role that S1PLs play in microbial pathogenicity was recently investigated in various strains of *Burkholderia*. These organisms do not encode SL biosynthesis genes but genome analysis revealed that *Burkholderia pseudomallei* K96243, a category B biothreat agent, encodes two homologous proteins (S1PL2021 and S1PL2025) that display moderate sequence identity to known eukaryotic and prokaryotic S1PLs.^277^,^278^ Both homologs were isolated and shown to catalyse S1PL-dependent conversion of S1P to 2*E*-HEX and PEA using a convenient fatty aldehyde dehydrogenase (FALDH) dependent coupled assay. The crystal structure of the *B. pseudomallei* S1PL2021 (Fig. 19, Table 1) was determined with the PLP cofactor bound. This enzyme displayed structural similarity to other S1PLs and, in the absence of a substrate or inhibitor, the authors propose a substrate-binding funnel that binds S1P. It will be interesting to screen inhibitors known to target the human S1PL as anti-microbial agents.^253^ The unexpected appearance of two copies of highly homologous S1PLs (possibly generated by gene duplication) in the *Burkholderia* was investigated by deletion studies, phenotypic analysis and macrophage survival. The S1PLs of *B. thailandensis* (BTH_II0309 and BTH_II0311) functionally complement S1PL-deficient yeast. Furthermore, S1PL knock out studies of both *Burkholderia* strains showed that the S1PLs are required for virulence in both *Galleria* moth and murine models of infection. These combined studies confirm a strong link between S1PL-dependent SL metabolism and virulence, but the reasons why *Burkholderia* in particular encode two S1PL copies requires further study.

### 5.2 Mechanism and inhibition of S1PL

**Fig. 20:**
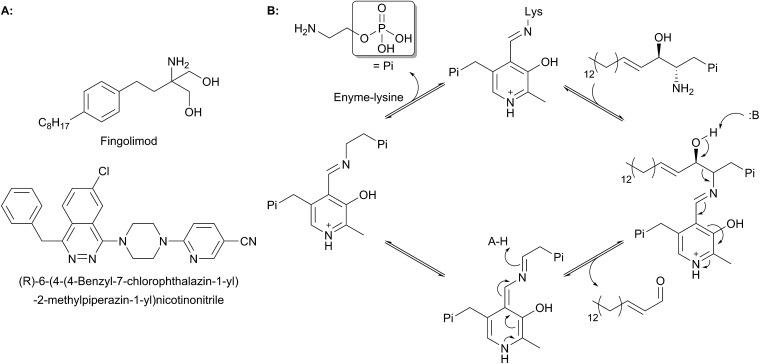
(A) Structure of FTY720 and the S1PL inhibitor (*R*)-6-(4-(4-benzyl-7-chlorophthalazin-1-yl)-2-methylpiperazin-1-yl)nicotinonitrile. (B) Proposed mechanism of S1P cleavage by S1PL, which involves the retro-aldol like cleavage of S1P.

Very few specific inhibitors of S1PL have been reported in the literature to date. Non-specific inhibitors of S1PL activity include metal ions (Ca^2+^, Zn^2+^), *N*-ethylmaleimide and iodoacetamide.^22^,^266^ Additionally, S1PL can also be inhibited by the 2d-, 3l-isomer of sphinganine and by sphinganine 1-phosphonate.^267^,^279^ The inhibitor FTY720 (an S1P receptor agonist derived from myriocin *via* a medicinal chemistry programme,^280^Fig. 20a), used as an immunomodulatory agent, can inhibit S1PL *in vitro*. Thymic extracts from mice following treatment with FTY720 also showed inhibited SPL activity.^281^ Two other functional SPL inhibitors identified include deoxypyridoxine and 2-acetyl-4-tetrahydroxybutylimidazole.^282^,^283^

Specific S1PL inhibitors have been published alongside the structure of human S1PL. Human S1PL was crystallised in the absence of the transmembrane region corresponding to residues 1–61.^284^ The overall structure is similar to that of StSPL and Dpl1p. The key active site residues (as discussed for StSPL) are listed in Table 3. Of note is the fact that the StSPL was crystallised in the presence of an SPL inhibitor, (*R*)-6-(4-(4-benzyl-7-chlorophthalazin-1-yl)-2-methylpiperazin-1-yl)nicotinonitrile, (Fig. 20a).^285^ This compound binds at the entrance of the tunnel leading from the surface of the protein to the active site, approximately 5 Å from the active site. This tunnel is composed of hydrophobic residues required for substrate binding and thus it is unsurprising that this is the binding site of the inhibitor.^284^

Since S1PL catalyses the retro-aldol like cleavage of S1P, a mechanism has been inferred by analogy to other type 1 PLP enzymes and from structural studies which have been performed.^271^ In the resting state, the PLP cofactor is bound to an active site lysine residue *via* an imine bond (Fig. 20b). Binding of the S1P substrate displaces this lysine to form a PLP-SP external aldimine. An as yet unknown active site base then deprotonates S1P at the C3 hydroxyl, resulting in cleavage of the C2/C3 σ bond, forming a PEA–PLP quinonoid and releasing 2*E*-HEX. Electron rebound from the quinonoid intermediate forms a PE-PLP external aldimine, before the PE is finally released *via* displacement by the active site lysine.

Some debate surrounds the identity of the base required to initiate the cleavage of S1P with a cysteine residue (Cys317 in human S1PL) proposed to perform this function.^12^ Early mutation experiments (C317S) performed before the crystal structure of S1PL was published, indicated that this residue was required for lyase activity.^12^ However, mutation of the equivalent cysteine in yeast Dpl1p (C344A) had no apparent effect on SPL activity in an *in vivo* yeast assay.^271^ Subsequent analysis of the human S1PL structure suggests that Cys317 is involved in binding of the PLP cofactor. As such, the identity of the active site base remains unknown, and may indeed be a function performed by the PLP binding lysine (as has been suggested in SPT).

### 5.3 Disease associated mutations in S1PL

Since S1PL plays such an important role in modulating cellular S1P levels it is not surprising to find that mutations of the enzyme result in various diseases. For example in a recent study of steroid-resistant nephrotic syndrome (SRNS) which causes 15% of the cases of chronic kidney disease, a genome analysis of SRNS-affected families revealed mutations in their S1PL genes.^286^ Biochemical studies explored the impact of such mutations on S1PL activity. Two such mutations (S346I and R222Q) are loss-of-function changes that lead to reduced protein levels and enzyme activity, as well as impaired degradation of long-chain sphingoid bases.

Lovric *et al.* also modelled the mutations of the human S1PL structure to gain insight into the molecular basis of human disease. These studies suggest that the R222Q mutation leads to a loss of two critical hydrogen bonding interactions at the dimer interface between Arg222 and the backbone Tyr250 and Ser249 residues. This increases the instability of the protein. The second S346I mutation is predicted to destabilise hydrogen bonding networks within the protein. Ser346 is buried deep within the S1PL structure and it is suggested that the sterically bulky isoleucine residue will cause steric clashes with nearby residues.

## 6 Conclusions

The vital role that SLs play in mammalian cell biology has been recognised for some time. More recently the importance of SL metabolism in various microbial species has gained increased attention. This then suggests that SLs are important players in mediating host/microbial interactions both in the context of beneficial, mutually-symbiotic relationships (*e.g.* the microbiome) but also in a harmful pathogenicity.

This review has discussed the biosynthesis and metabolism of LCBs highlighting studies of enzymes which have been investigated with detailed structural and mechanistic methods. In the most simple microbial systems where the enzymes are soluble, cytoplasmic and available in highly homogeneous recombinant forms (*e.g. S. paucimobilis* SPT), high resolution crystal structures have provided insight into the molecular mechanism of these enzyme-catalysed conversions. They have also provided models for the more complicated, membrane-bound, multi-subunit complexes which have been identified in eukaryotes. These studies have also allowed us to hypothesise on the impacts of genetic mutation of SL-enzymes (*e.g.* HSAN1) at the molecular level. A key, over-arching theme in SL metabolism is that the cell must maintain a balanced pool of sphingolipidome components that allows it to generate SLs from the l-serine and fatty acid pools in times of need, but also degrade SLs and ceramides since these are potentially toxic if allowed to accumulate. One interesting area to investigate is the roles that are played by the so-called deoxy-SLs (derived from l-alanine and glycine) which represent biomarkers for diseases such as diabetes.^287^,^288^

It would make sense to tightly regulate SL metabolism by controlling the catalytic activity of key enzymes in the pathway. These include those enzymes discussed here (SPT, SK, S1PL) and others that space prohibited us from reviewing (*e.g.* the ceramide synthase (CS) and the sphingomyelin synthase (SMS) families and associated degradation enzymes^289^–^291^). The increasingly complicated nature of the multi-subunit SPT, the first enzyme in all eukaryotes that make SLs, has revealed it is much more complex than initially thought and is associated with activators and regulators.

The complex nature of the enzymes involved in SL metabolism has not prevented drug companies from targeting this pathway in the hope of generating new therapeutics.^176^,^177^ This is exemplified by Fingolimod, a clinical success in the treatment of MS, derived from myriocin by a medicinal chemistry programme.^292^ Natural products have key roles in SL metabolism. Detailed structural and biochemical studies will aid in the discovery of new interventions, for which natural products will undoubtedly provide lead compounds.

## 7 Conflicts of interest

There are no conflicts to declare.
